# Interleukin 33-mediated inhibition of A-type K^+^ channels induces sensory neuronal hyperexcitability and nociceptive behaviors in mice

**DOI:** 10.7150/thno.69320

**Published:** 2022-02-14

**Authors:** Yiru Wang, Xinyi Wang, Renfei Qi, Ying Lu, Yu Tao, Dongsheng Jiang, Yufang Sun, Xinghong Jiang, Chunfeng Liu, Yuan Zhang, Jin Tao

**Affiliations:** 1 Department of Geriatrics & Clinical Research Center of Neurological Disease, the Second Affiliated Hospital of Soochow University, Suzhou 215004, P.R. China.; 2 Department of Physiology and Neurobiology, Medical College of Soochow University, Suzhou 215123, P.R. China.; 3 Institute of Regenerative Biology and Medicine, Helmholtz Zentrum München, Munich 81377, Germany.; 4 Department of Neurology and Clinical Research Center of Neurological Disease, the Second Affiliated Hospital of Soochow University, Suzhou 215004, P.R. China.; 5 Centre for Ion Channelopathy, Soochow University, Suzhou 215123, P.R. China.; 6 Jiangsu Key Laboratory of Neuropsychiatric Diseases, Soochow University, Suzhou 215123, P.R. China.

**Keywords:** interleukin 33, A-type K^+^ channel, dorsal root ganglion neurons, pain

## Abstract

**Background:** Interleukin-33 (IL-33) has been implicated in nociceptive pain behaviors. However, the underlying molecular and cellular mechanisms remain unclear.

**Methods:** Using electrophysiological recording, immunoblot analysis, immunofluorescence labeling, reverse transcription-PCR, siRNA-mediated knockdown approach and behavior tests, we determined the role of IL-33 in regulating sensory neuronal excitability and pain sensitivity mediated by A-type K^+^ channels.

**Results:** IL-33 decreased A-type transient outward K^+^ currents (*I*_A_) in small-sized DRG neurons in a concentration-dependent manner, whereas the delayed rectifier currents (*I*_DR_) remained unaffected. This IL-33-induced *I*_A_ decrease was dependent on suppression of the tumorigenicity 2 (ST2) receptor and was associated with a hyperpolarizing shift in the steady-state inactivation. Antagonism of Syk abrogated the IL-33-induced *I*_A_ response, while inhibition of JAK2 and PKA elicited no such effect. Exposure of DRG cells to IL-33 increased the activity of Akt, but surprisingly, neither Akt nor PI3K influenced the IL-33-induced *I*_A_ response. IL-33 increased the level of phosphorylated p38 mitogen-activated protein kinase (MAPK). Chemical inhibition of p38 and genetic siRNA knockdown of p38 beta (p38β), but not p38α, abrogated the *I*_A_ response induced by IL-33. Moreover, IL-33 increased neuronal excitability of DRG neurons and facilitated peripheral pain sensitivity in mice; both of these effects were occluded by *I*_A_ blockade.

**Conclusions:** Our present study reveals a novel mechanism by which IL-33/ST2 suppresses *I*_A_ via a Syk-dependent p38β signaling pathway. This mechanism thereby increases DRG neuronal excitability and pain sensitivity in mice. Targeting IL-33/ST2-mediated p38β signaling may represent a therapeutic approach to ameliorate pain behaviors.

## Introduction

Interleukin-33 (IL-33), a member of the interleukin-1 (IL-1)-associated cytokine family, is widely expressed in the mammalian brain and peripheral nervous tissues 1, 2. Two different suppressor of tumorigenicity 2 (ST2) receptor isoforms, soluble ST2 (sST2) and transmembrane ST2, have been identified as endogenous receptors for IL-33. While ST2 mediates the effects of IL-33, sST2, a decoy receptor, limits IL-33 activity 3. Acting through the membrane ST2 receptor, IL-33 fulfils a variety of important biological functions in the pathogenesis of neurological diseases such as Alzheimer's disease, stroke and multiple sclerosis 4-7. Recent evidence has also suggested a functional role of IL-33/ST2 in nociceptive pain behaviors 8-13. For instance, it has been demonstrated that IL-33 and its receptor ST2 in the DRG were upregulated in a rat model of spared nerve injury (SNI), and intrathecal injection of either an IL-33 antagonist or a neutralizing antibody to ST2 alleviated mechanical allodynia 13, 14. Moreover, genetic deletion of ST2 resulted in amelioration of pain hypersensitivity in a mouse model of gout 15. Further evidence to support this hypothesis came from the clinical finding that serum IL-33 levels were significantly higher in patients with gout than in healthy controls 16. Nevertheless, the underlying molecular and cellular mechanisms of IL-33 participating in peripheral nociceptive responses are not fully understood.

Alterations in peripheral sensory neuronal excitability can directly affect nociceptive behaviors 17. Voltage-gated K^+^ channels (Kv) are pivotal components of action potential generation and propagation 18, and Kv currents have been grouped as A-type transient outward currents (*I*_A_) or delayed rectifier currents (*I*_DR_) in primary sensory neurons 19-21. *I*_A_ can be defined by sensitivity to 4-aminopyridine (4-AP) and with characteristics of rapid activation and inactivation 22-24. These properties contribute critically to action potential repolarization and have been widely implicated in dendritic integration and pain plasticity 22. Molecular, genetic, and functional analyses have identified a pivotal role of *I*_A_ in amplifying peripheral nociceptive processing and central sensitization promotion 21, 22, 25. Moreover, it has also been shown that nerve injury might induce the downregulation of *I*_A_, leading to increased excitability of nociceptive neurons, thereby increasing responsiveness to nociceptive stimuli 26. Therefore, manipulation of *I*_A_ may affect neuronal excitability and the subsequent transmission of nociceptive signals and has been considered a potential therapeutic strategy for the treatment of pain.

In the present study, we identified a critical role of IL-33/ST2 in the regulation of *I*_A_ and elucidated the underlying molecular components of the signaling that elicit the nociceptive response of IL-33. We demonstrated that IL-33 binding to ST2 triggers the activation of Syk and downstream p38β. This signaling-mediated suppression of *I*_A_ induces neuronal hyperexcitability of DRG neurons and contributes to pain hypersensitivity in mice.

## Materials and methods

### Isolation of DRG neurons

All procedures used in this study were approved by the Animal Care and Use Committee of Soochow University and are in accordance with the National Institutes of Health Guidelines for the Care and Use of Laboratory Animals. Mice were housed with three to five per cage on a 12 h/12 h light/dark cycle with food and water available *ad libitum*. Every effort was made to minimize both the number of animals used and their suffering. DRGs were removed from the L4-6 lumbar segments of these mice (ICR, males, 6-8 weeks) and enzymatically dissociated as described in our previous studies 27-29. In brief, after trimming the connective tissue and nerve fibers, the ganglia were incubated with collagenase (1.5 mg/mL, Roche) at 37 °C for 30 min and then digested with 0.25% trypsin for 10 min at 37 °C. Cells were triturated with fired-polished Pasteur pipettes. DRG cells were plated in a droplet of growth medium on glass coverslips coated with Matrigel (Merck). Coverslips were kept for 2 h in an incubator before being flooded with neurobasal medium supplemented with 2% B27 (Gibco). DRG neurons were used for patch clamp experiments within 3-6 h of plating. We sorted the DRG neurons into three groups: small- (soma diameter < 30 μm), medium- (soma diameter 30-45 μm), and large-sized (soma diameter > 45 μm) neurons 30, and limited the recordings to small-sized DRG neurons.

### Electrophysiology

Whole-cell voltage- and current-clamp recordings were conducted at room temperature (23 ± 1 °C) using a MultiClamp 700B (Molecular Devices) as described in our previous studies 27, 28, 31. Data were low-pass filtered at 2 kHz and sampled at 10 kHz. Series resistance was compensated at least 80%, and leak subtraction was performed. The patch pipettes were pulled from borosilicate capillaries (Sutter Instruments) and had a resistance of 3-5 MΩ when filled with pipette solution. The recording chamber was continuously superfused (3-4 mL/min). For voltage-gated K^+^ channel (Kv) current recordings, the external solution was composed of (in mM) 5 KCl, 1 MgCl_2_, 0.03 CaCl_2_, 150 choline-Cl, 10 HEPES, and 10 glucose adjusted to pH 7.4 with KOH; osmolarity, 310 mOsm with sucrose. The pipette solution was composed of (in mM) 140 KCl, 1 MgCl_2_, 0.5 CaCl_2_, 10 HEPES, 5 EGTA, 0.3 Na-GTP, and 3 Mg-ATP adjusted to pH 7.4 with KOH; osmolarity, 295 mOsm with sucrose. For voltage-gated Ca^2+^ channel (Cav) current recordings, the external solution was composed of (in mM) 140 tetraethylammonium chloride (TEA-Cl), 5 BaCl_2_, 0.5 MgCl_2_, 5 CsCl, 10 HEPES, and 5.5 glucose adjusted to pH 7.35 with TEA-OH; osmolarity, 305 mOsm with sucrose. The pipette solution was composed of (in mM): 110 CsCl, 25 HEPES, 0.3 Na-GTP, 4 Mg-ATP, and 10 EGTA adjusted to pH 7.4 with CsOH; osmolarity, 295 mOsm with sucrose. For current-clamp and Nav current recordings, the external solution was composed of (in mM): 128 NaCl, 2 KCl, 2 CaCl_2_, 2 MgCl_2_, 25 HEPES, and 30 glucose adjusted to pH 7.4 with NaOH; osmolarity, 305 mOsm with sucrose. The pipette solution was composed of (in mM): 110 KCl, 25 HEPES, 10 NaCl, 0.3 Na-GTP, 4 Mg-ATP, and 2 EGTA adjusted to pH 7.4 with KOH; osmolarity, 295 mOsm with sucrose. Compounds were delivered to the patched neurons with an air-pressure microinjector (Pneumatic PicoPump, PV830). For whole-cell recordings in which neurons were intracellular dialyzed with compounds, the path pipettes had a resistance of 2-3 MΩ, and recordings were initiated 5 min after breaking the patch.

### Reverse transcription-PCR (RT-PCR)

Total RNA was extracted from mouse lumbar (L4-6) DRGs using an RNeasy Plus Mini kit (Qiagen) as described previously 32, 33. RNA was reverse-transcribed using SuperScript^TM^ III reverse transcriptase (Thermo Scientific). The sequences of the primers (positions according to ST2, NM_001025602.3) designed by Primer 5.0 software were as follows: forward 5′-ACCATACATA AAAAGCCGCCAAGC-3′, and reverse 5′-ATCTGCCACAGGACATCAGC CAAG-3′. PCR analysis was repeated in triplicate with the same samples to confirm the reproducibility of the results.

### Immunoblot analysis

Immunoblot analysis was performed as described in our previous studies 27, 32, 34. In brief, samples containing 25 μg of protein were separated on SDS-polyacrylamide gel electrophoresis, electroblotted onto polyvinylidene difluoride membranes (Merk Millipore), and probed with antibodies against ST2/IL-33R (rabbit, 1:1000; Novus Biologicals, Cat. No. NBP2-53096), Syk (rabbit, 1:1000; Cell Signaling Technology, Cat. No. #2712), *p*-Syk (rabbit, 1:1000; Cell Signaling Technology, Cat. No. #2710), JAK2 (rabbit, 1:1000; Cell Signaling Technology, Cat. No. #3230), *p*-JAK2 (rabbit, 1:500; Cell Signaling Technology, Cat. No. #3771), *p*-p38 (rabbit, 1:1000; Cell Signaling Technology, Cat. No. #4511), p38 (rabbit, 1:1000; Cell Signaling Technology, Cat. No. #8690), *p*-ERK (rabbit, 1:1000; Cell Signaling Technology, Cat. No. #4370), ERK (rabbit, 1:1000; Cell Signaling Technology, Cat. No. #4695),* p*-Akt (rabbit, 1:800; Cell Signaling Technology, Cat. No. #4060), Akt (rabbit, 1:800; Abcam, Cat. No. ab8805), *p*-JNK (rabbit, 1:1000; Cell Signaling Technology, Cat. No. #4668), JNK (rabbit, 1:1000; Cell Signaling Technology, Cat. No. #9252), p38α (rabbit, 1:1000; Cell Signaling Technology, Cat. No. #9218), p38β (rabbit, 1:1000; ProteinTech Group, Cat. No. 17376-1-AP) and β-tubulin (rabbit, 1:1000; ProteinTech Group, Cat. No. 10094-1-AP). The blots were washed and then detected with the appropriate horseradish peroxidase-conjugated secondary antibody (Cell Signaling Technology). Signals were visualized with enhanced chemiluminescence (Thermo Fisher Scientific) and detected with a ChemiDoc XRS System (Bio-Rad Laboratories). The intensities of individual bands were quantified using Quantity One (Bio-Rad Laboratories). The full-length blots are presented in the Supplementary Data.

### Immunofluorescence staining

Immunostaining was performed as described previously 27, 34. Briefly, DRGs were cut by a cryostat (Leica CM1950). The sections were permeabilized with 0.2% Triton X-100 for 0.5 h, blocked with 5% normal goat serum, and incubated overnight at 4 °C with antibodies against ST2/IL-33R (rabbit, 1:400; Sigma, Cat. No. PRS3363), NeuN (mouse, 1:500; Cell Signaling Technology, Cat. No. #94403), glutamine synthetase (GS, mouse, 1:500; Abcam, Cat. No. ab64613), Neurofilament 200 (NF200, mouse, 1:500; Abcam, Cat. No. ab215903), calcitonin gene-related peptide (CGRP, mouse, 1:500; Abcam, Cat. No. ab81887), Syk (mouse, 1:500; Thermo Fisher Scientific, Cat. No. MA5-17207), JAK2 (mouse, 1:300; Santa Cruz Biotechnology, Cat. No. sc-390539), Akt (mouse, 1:300; ProteinTech Group, Cat. No. 60203-2-lg), PKA (mouse, 1:200; Thermo Fisher Scientific, Cat. No. MA5-37857), p38β (mouse, 1:300; Santa Cruz Biotechnology, Cat. No. sc-390984) and FITC-conjugated mouse anti-isolectin B4 (IB4, 1:500; Sigma, Cat. No. L2895). To increase the specificity of the ST2 antibody, the ST2 antibody was preadsorbed against its blocking peptide (Sigma, Cat. No. SBP3363) prior to incubation with DRG sections, by mixing the ST2 antibody with a five-fold (by weight) of the blocking peptide. After washing with PBS, the sections were incubated for 1 h at room temperature with the appropriate secondary antibodies: Alexa Fluor 555 goat anti-rabbit (1:300, Cell Signaling Technology, Cat. No. #4413) and Alexa Fluor 488 goat anti-mouse (1:300, Cell Signaling Technology, Cat. No. #4408). All images were acquired using a Nikon 104c fluorescence microscope. Negative control sections (no exposure to the primary antisera) were processed concurrently for all immunostaining studies. No significant staining was detected in these samples.

### Behavioral test

Animals were habituated to the testing environment daily for at least 2 d before baseline testing. All behavioral experiments were performed by individuals who were blinded to the treatments. Mice were placed in cages on an elevated metal mesh floor and acclimated for at least 30 min before the first assessment. To test mechanical sensitivity, the plantar surface of the hindpaw was stimulated with an ascending series of *von* Frey hairs (0.02-2.56 g, Stoelting), and the 50% paw withdrawal thresholds (PWTs) were determined using Dixon's up-down method 35. Thermal sensitivity, expressed as paw withdrawal latency (PWL), was assessed using a commercially available Thermal Plantar Test Instrument (Hargreave's Method, Ugo Basile 37370-001) to measure the response to an infrared heat stimulus as described previously 27. The baseline latencies were adjusted to 10-14 s, with a maximum of 18 s as the cut-off to prevent potential injury. The latencies were averaged over three trials separated by a 5 min interval. Drug or vehicle was injected subcutaneously into the plantar surface of the hind paw in a volume of 10 μl. All solutions were pH balanced to 7.4 to avoid skin irritation.

### SiRNA administration

5′-Cholesteryl-modified and 2′-O-methyl-modified siRNA (ST2 siRNA: 5′-UUUAGCAU GAUCUCUGGCG-3′; p38β siRNA: 5′-CGCCAGAGAUCAUGCUAAA-3′) labelled with Cy3 and scrambled siRNAs were purchased from RiboBio Biological Technology (Guangzhou). The siRNA sequences were subjected to BLAST analysis to minimize any potential off-target effects. SiRNA was dissolved in RNase-free water at 1 µg/µl as a stock solution and mixed with the transfection reagent polyethyleneimine (PEI; Fermentas Inc), an organic polyamine polymer, and normal saline before use. PEI is a synthetic polymer that has been successfully used as a siRNA delivery vehicle during gene silencing applications 36-38. Intrathecal injections were made with a 30 G needle between the L5 and L6 intervertebral space to deliver the siRNA-PEI complexes. Treatment was repeated every 12 h thereafter for a total of 3 d. The siRNA efficacy on ST2 or p38β expression in DRGs was analyzed by immunoblotting 12 h after the last siRNA injection. Small DRG neurons with red fluorescence under an inverted phase-contrast microscope (Nikon Eclipse Ti-S) 3 d after injection were subjected to whole-cell patch-clamp recordings.

### Drugs and reagents

All drugs were obtained from Sigma-Aldrich unless otherwise indicated. IL-33 was obtained from Novus Biologicals. The ST2 neutralizing antibody was obtained from R&D System. The soluble form of ST2 (sST2) was obtained from Cohesion Biosciences. KT-5720 was obtained from Tocris. GS9973 and R406 were obtained from Selleck. JX-401 was obtained from Abcam. Stock solutions of KT-5720, AG490, R406, GS9973, LY294002, Akt inhibitor III, SB203580, SB202474 and JX401 were prepared in dimethyl sulfoxide (DMSO). The final concentration of DMSO in the bath (maximum of 0.05%) had no functional effects on the currents measured.

### Statistical analysis

All data are expressed as the mean ± S.E.M. Off-line evaluation was performed using Clampfit 10.2 and GraphPad Prism v7.0. Statistical significance was assessed by one-way ANOVA followed by a* post hoc* Bonferroni test for multiple comparisons between groups. Paired or two-sample Student's t tests were used when comparisons were restricted to two means. Behavioral data were assessed by two-way repeated-measures ANOVA with a *post hoc* Bonferroni test. Error probabilities of p < 0.05 were considered statistically significant. The voltage-dependent activation and steady-state inactivation of I_A_ were fitted to the Boltzmann function. The IL-33 dose-response data were fitted to the Hill equation by a nonlinear regression algorithm to yield the parameters describing the dose of IL-33 that elicited the half-maximal inhibitory response (IC_50_) and the Hill coefficient (*n*).

## Results

### IL-33 selectively decreases *I*_A_ in small DRG neurons

**Figure 1:**
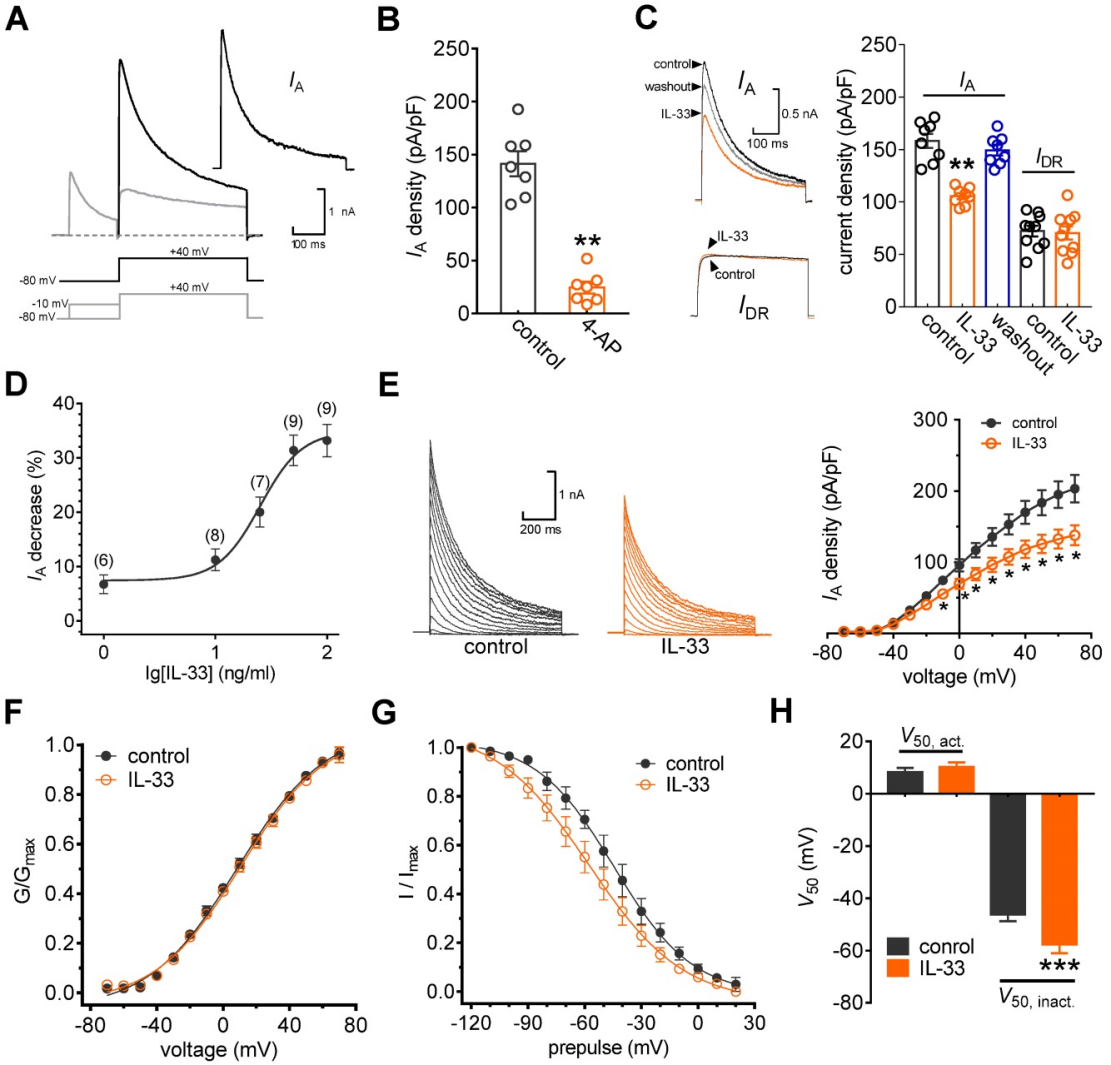
** IL-33 suppresses *I*_A_ in DRG neurons. A,** *I*_A_ isolation. *I*_A_ was acquired after off-line subtraction of the noninactivating component of the current remaining after a brief prepulse of -10 mV. Stimulation protocols for *I*_A_ isolation are shown in the panel below. *Inset*, subtracted *I*_A_. **B,** Summary data showing the effect of 4-AP (5 mM) on *I*_A_ density (*n* = 7 cells). **p < 0.01* vs.* control, paired *t* test. The *I*_A_ density was measured from the ratio of the peak current amplitude to the membrane capacity (pA/pF). **C,** Example traces (*left*) and summary data (*right*) indicating that 50 ng/mL IL-33 decreases *I*_A_ (*n* = 8 cells) but does not affect *I*_DR_ (*n* = 10 cells). **p < 0.01* vs.* control, paired t test. **D,** Dose-response curve indicating the percent change in *I*_A_. Data were fitted to a sigmoidal *Hill* function. The number of cells tested at each concentration of IL-33 is shown in brackets. **E,** example traces (*left*) and mean current/voltage plot of *I*_A_ density *vs.* test potential (*right*) for the control and 50 ng/mL IL-33 treatment (*n* = 15 cells). *p < 0.05 *vs.* control, one-way ANOVA with a Bonferroni post hoc test. **F-G,** voltage dependence of activation (*F*, *n* = 13 cells) and steady-state inactivation (*G*, *n* = 10 cells) curves before and after 50 ng/mL IL-33 application. To assess voltage-dependent activation, voltage commands were applied (ranging from -70 mV to +70 mV) in 10 mV increments. The steady-state inactivation curve was determined by conditioning a prepulse varying from -120 mV to +20 mV in 10 mV increments followed by a test pulse to +40 mV. **H,** Summary data showing the effect of 50 ng/mL IL-33 on *V*_50_ activation (*V*_50, act_) and inactivation curves (*V*_50, inact_). ***p < 0.001 *vs.* control, paired t test.

In this study, we restricted the whole-cell patch clamp recordings to small-sized neurons (soma diameter < 30 μm), as they are the primary participants in peripheral nociceptive processing 17, 27, 28. Two major subtypes of Kv currents, A-type transient outward currents (I_A_s) and delayed rectifier currents (I_DR_s), have been characterized in these nociceptive neurons 20, 39. Thus, we first isolated these two kinetically different whole-cell currents. As shown in Figure 1A, a total outward current was elicited by a command potential of +40 mV from a holding potential of -80 mV. The typical currents recorded from these neurons exhibit a fast, inactivating component followed by a sustained current. A 150 ms prepulse to -10 mV allowed the transient channels to inactivate, leaving only the sustained current (*I*_DR_). *I*_A_ was then isolated by subtracting the *I*_DR_ from the total current (Figure 1A). This I_A_ was blocked by 5 mM 4-AP, further confirming the effective isolation of I_A_ (decrease of 83.7 ± 5.3%, Figure 1B). Application of IL-33 (50 ng/mL) to small neurons markedly reduced I_A_ by 31.9 ± 2.8%, while I_DR_ remained unaffected (decrease of 2.4 ± 0.9%, Figure 1C). The reduction in I_A_ was partially reversible upon IL-33 washout (Figure 1C). Testing the effects of varying concentrations of IL-33 on I_A_ revealed that the inhibitory effects were dose-dependent (Figure 1D). The IC_50_ determined for the inhibition of I_A_ by IL-33 was 27.1 ng/mL. Next, we characterized the underlying biophysical basis of the IL-33-induced I_A_ response. Peak I_A_ decreased significantly in response to bath application of 50 ng/mL IL-33 at all potentials above -10 mV (Figure 1E). Moreover, IL-33 did not affect the voltage dependence of the activation properties (*V*_50, act_) (Figure 1F-H) but caused a significant hyperpolarizing shift in the half-inactivation potential (*V*_50, inact_) of I_A_ (~11.3 mV, Figure 1G-H).

### ST2 mediates the IL-33-induced *I*_A_ response

**Figure 2:**
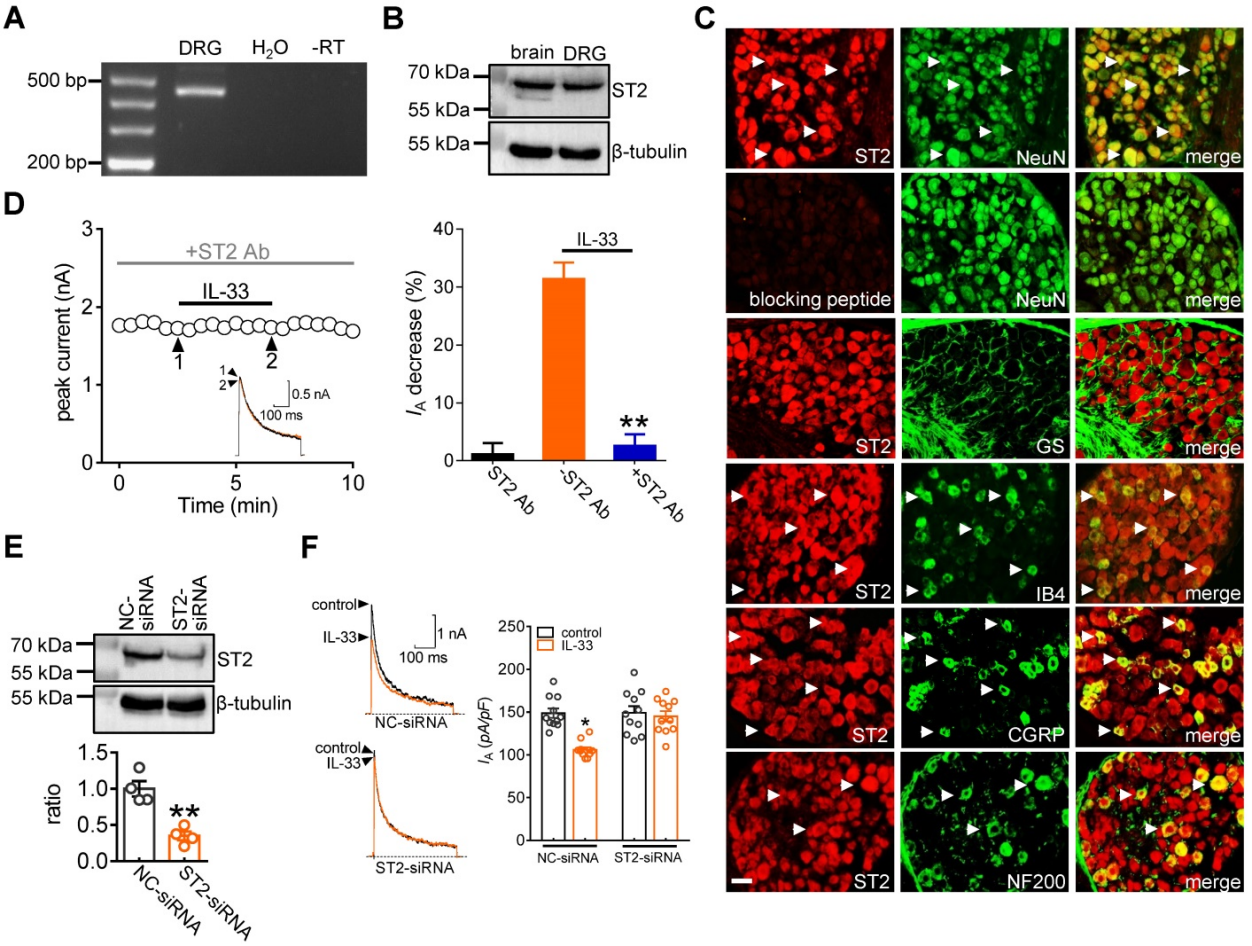
**ST2 mediates the IL-33 response on *I*_A_. A,** Detection of ST2 transcripts in mouse DRGs. Neither the reverse-transcription negative control (without reverse transcriptase, -RT) nor nontemplate negative control (-H_2_O) showed a signal. **B,** Immunoblot analysis of ST2 protein abundance in mouse DRGs. Blots are representative of three independent experiments with β-tubulin serving as a loading control. **C,** Colabeling (white arrows) of ST2 and NeuN, GS, CGRP, IB_4_ and NF200 in mouse DRG sections. Pre-incubation of ST2 antibody with excessive ST2 blocking peptide served as the specificity control of ST2 antibody. Scale bar, 50 µm. **D,** Time course of *I*_A_ changes (*left*) and bar graph (*right*) demonstrating that pretreating DRG neurons with an ST2 neutralizing antibody (ST2 Ab, 2 µg/mL) prevented the IL-33-induced* I*_A_ decrease (*n* = 9 cells). The application of 2 µg/mL ST2 Ab alone did not affect *I*_A_ (*n* = 7 cells). Arabic numerals indicate the points utilized for example current traces. **p < 0.01 *vs.* IL-33 without ST2 Ab, paired t test. **E,** Immunoblot analysis of ST2 protein abundance in the control siRNA (NC-siRNA) and ST2 siRNA-treated (ST2-siRNA) groups. Blots are representative of three independent experiments with β-tubulin serving as a loading control. **p < 0.01 *vs.* NC-siRNA, unpaired t test. **F,** Bar graph indicating that treatment with ST2-siRNA (*n* = 12 cells), but not NC-siRNA (*n* = 11 cells), abrogated the 50 ng/mL IL-33-induced *I*_A_ decrease. *p < 0.05 *vs.* control + NC-siRNA, one-way ANOVA with a Bonferroni post hoc test.

It has been shown that IL-33 fulfils a variety of biological functions via the membrane suppression of tumorigenicity 2 receptor (ST2) 3, 6. Thus, we determined whether ST2 participates in the IL-33-induced* I*_A_ decrease. RT-PCR analysis revealed that ST2 transcripts were present in mouse DRGs (Figure 2A & Figure S1). Further immunoblot analysis of DRG cell lysates revealed the protein expression of ST2 (Figure 2B & Figure S2). Moreover, immunostaining of intact mouse DRGs indicated that ST2 was coexpressed with NeuN but not in glutamine synthetase (GS)-labeled cells (Figure 2C). To ensure the high specificity of the ST2 antibody, the ST2 antibody was preadsorbed against its blocking peptide prior to incubation with DRG sections. Pre-incubation of the ST2 primary antibody with the recombinant blocking peptide abrogated the detection of ST2 in DRGs by immunofluorescence assays (Figure 2C). Further double immunofluorescence staining showed that ST2 was expressed mostly in calcitonin gene-related peptide (CGRP)-labelled peptidergic neurons and isolectin B4 (IB_4_)-labelled nonpeptidergic neurons but to a much lesser extent in neurofilament-200 (NF200)-labelled myelinated neurons (Figure 2C). Next, we determined the relative participation of ST2 in the IL-33-induced *I*_A_ decrease. Application of the specific ST2 neutralizing antibody alone (2 μg/mL) had no significant effect on *I*_A_ (decrease of 1.1 ± 1.9%), while pretreating DRG neurons with an ST2 neutralizing antibody completely abolished the decrease in *I*_A_ induced by 50 ng/mL IL-33 (decrease of 2.5 ± 1.9%) (Figure 2D), indicating the participation of ST2 in the IL-33-induced *I*_A_ response. As complimentary support of our hypothesis, we further examined the effect of IL-33 on *I*_A_ in ST2-silenced DRG neurons. Immunoblot analysis indicated that the protein expression level of ST2 was markedly decreased in the ST2 siRNA-treated groups (Figure 2E & Figure S3). Knockdown of ST2 abrogated the IL-33-induced *I*_A_ decrease in small DRG neurons (decrease of 3.9 ± 1.6%, Figure 2F).

### Syk is involved in the IL-33/ST2-mediated *I*_A_ response

**Figure 3:**
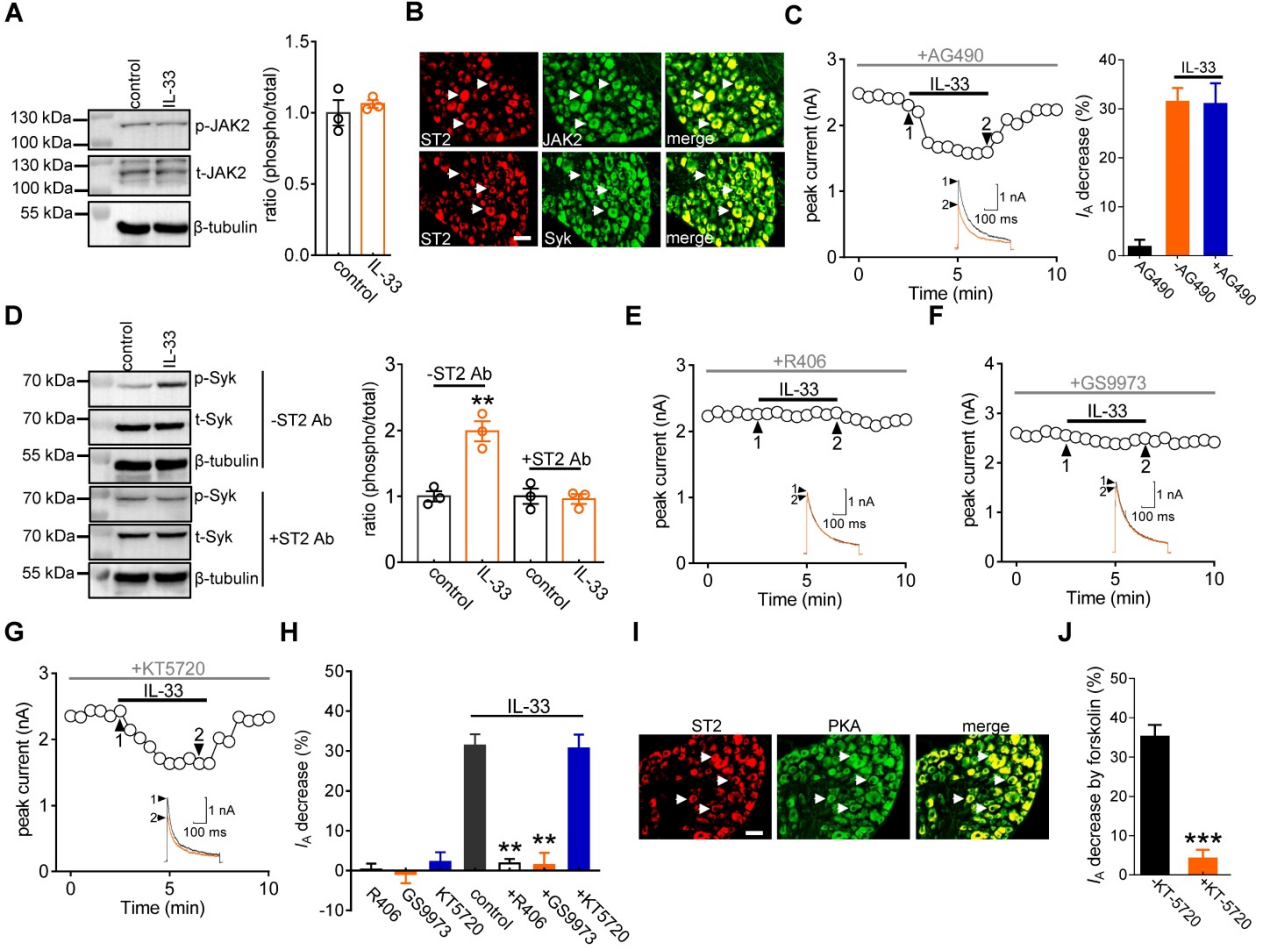
** The IL-33-induced *I*_A_ decrease requires Syk. A,** effects of 50 ng/mL IL-33 on the protein abundance of phospho-JAK2 (*p*-JAK2) or total JAK2 (*t*-JAK2). Blots are representative of three independent experiments with β-tubulin serving as a loading control. **B,** colabeling (white arrows) of ST2 and JAK2 and Syk in mouse DRG sections. Scale bar, 50 µm. **C,** time course of *I*_A_ changes (*left*) and bar graph (*right*) showing that pretreatment of DRG neurons with AG490 (10 µM) did not affect the IL-33-induced* I*_A_ response (*n* = 8 cells). Application of 10 µM AG490 alone did not affect *I*_A_ (*n* = 7 cells). Arabic numerals indicate the points utilized for the example current traces. **D,** effects of 50 ng/mL IL-33 on* p*-Syk protein abundance in the presence or absence of the ST2 neutralizing antibody (ST2 Ab, 2 µg/mL) in DRG cells. Blots are representative of three independent experiments with β-tubulin serving as a loading control. **p < 0.01* vs.* control, unpaired t test. **E-G,** time course of the *I*_A_ changes showing that pretreating DRG neurons with R406 or GS9973, but not KT-5720, prevented the IL-33-induced* I*_A_ response. Arabic numerals indicate the points utilized for the example current traces. **H,** bar graph showing the effect of 50 ng/mL IL-33 on *I*_A_ in cells pretreated with R406 (1 µM, *n* = 11 cells), GS9973 (10 µM,* n* = 8 cells), and KT-5720 (1 µM,* n* = 9 cells). Application of 1 µM R406 (*n* = 7 cells), 10 µM GS9973 (*n* = 6 cells) or 1 µM KT-5720 (*n* = 7 cells) alone had no significant effect on *I*_A_. **p < 0.01* vs.* control, paired t test. **I,** colabeling (white arrows) of ST2 and PKA in mouse DRG sections. Scale bar, 50 µm. **J,** bar graph demonstrating the effect of 20 µM forskolin on *I*_A_ in the presence (*n* = 7 cells) or absence (*n* = 5 cells) of KT-5720 (1 µM). ***p < 0.001 *vs.* forskolin without KT-5720, paired t test.

Previous studies have demonstrated that JAK2/STAT3 signaling is involved in IL-33/ST2-mediated biological responses 40, 41. Immunoblot analysis confirmed the protein expression of JAK2 in DRG cells (Figure 3A & Figure S4). Moreover, immunostaining of intact mouse DRGs indicated that JAK2 was coexpressed with ST2 (Figure 3B). Interestingly, in mouse DRG cells, the protein expression levels of total JAK2 (*t*-JAK2) and phosphorylated JAK2 (*p*-JAK2) were unaffected by 50 ng/mL IL-33 application (Figure 3A & Figure S4). Consistent with this, preincubating DRG neurons with the JAK2-specific inhibitor AG490 (10 μM) did not influence the inhibitory effect of IL-33 on *I*_A_ (decrease of 30.9 ± 4.2%, Figure 3C). It has been demonstrated that Syk is a nonreceptor tyrosine kinase involved in several cytokine signaling pathways 42, 43. Immunoblot analysis of DRG cell lysates revealed the protein expression of Syk (Figure 3D & Figure S5), and immunofluorescence staining showed that Syk was coexpressed with ST2 (Figure 3B). We thus examined whether IL-33 action in DRG neurons requires Syk activation. Exposure of DRG cells to IL-33 markedly increased the expression level of phosphorylated Syk (*p*-Syk), while the total Syk (*t*-Syk) level remained unaffected (Figure 3D & Figure S5). IL-33-induced Syk activation was prevented by pretreating cells with the ST2 neutralizing antibody (2 μg/mL) (Figure 3D & Figure S5), indicating the involvement of ST2-dependent Syk signaling. Furthermore, pretreating DRG neurons with the small-molecule Syk inhibitor R406 (1 µM) abrogated the IL-33-induced decrease in *I*_A_ (decrease of 1.8 ± 1.1%, Figure 3E-H). Similar results were obtained with another highly selective Syk inhibitor, GS9973. Pretreatment of DRG neurons with 10 μM GS9973 eliminated the *I*_A_ response induced by 50 ng/mL IL-33 (decrease of 1.4 ± 3.1%, Figure 3F-H). Thus, Syk activation is involved in the IL-33/ST2-induced *I*_A_ response. Protein kinase A (PKA) activity can be regulated by Syk 44 and might participate in the regulation of *I*_A_ 28. We therefore determined whether the Syk-mediated *I*_A_ response induced by IL-33/ST2 was PKA-dependent. Pretreating DRG neurons with KT-5720 (1 μM), a PKA-specific inhibitor, did not affect the IL-33-mediated *I*_A_ response (decrease of 30.7 ± 3.4%; Figure 3G-H). PKA was coexpressed with ST2 in mouse DRG neurons (Figure 3I), and KT5720 (1 μM) used in this study was found to be effective in PKA inhibition since pretreatment with KT-5720 completely prevented the forskolin (20 μM)-induced *I*_A_ decrease in DRG neurons (decrease of 4.1 ± 2.2%, Figure 3J).

### The IL-33-induced *I*_A_ decrease requires p38 MAPK

**Figure 4:**
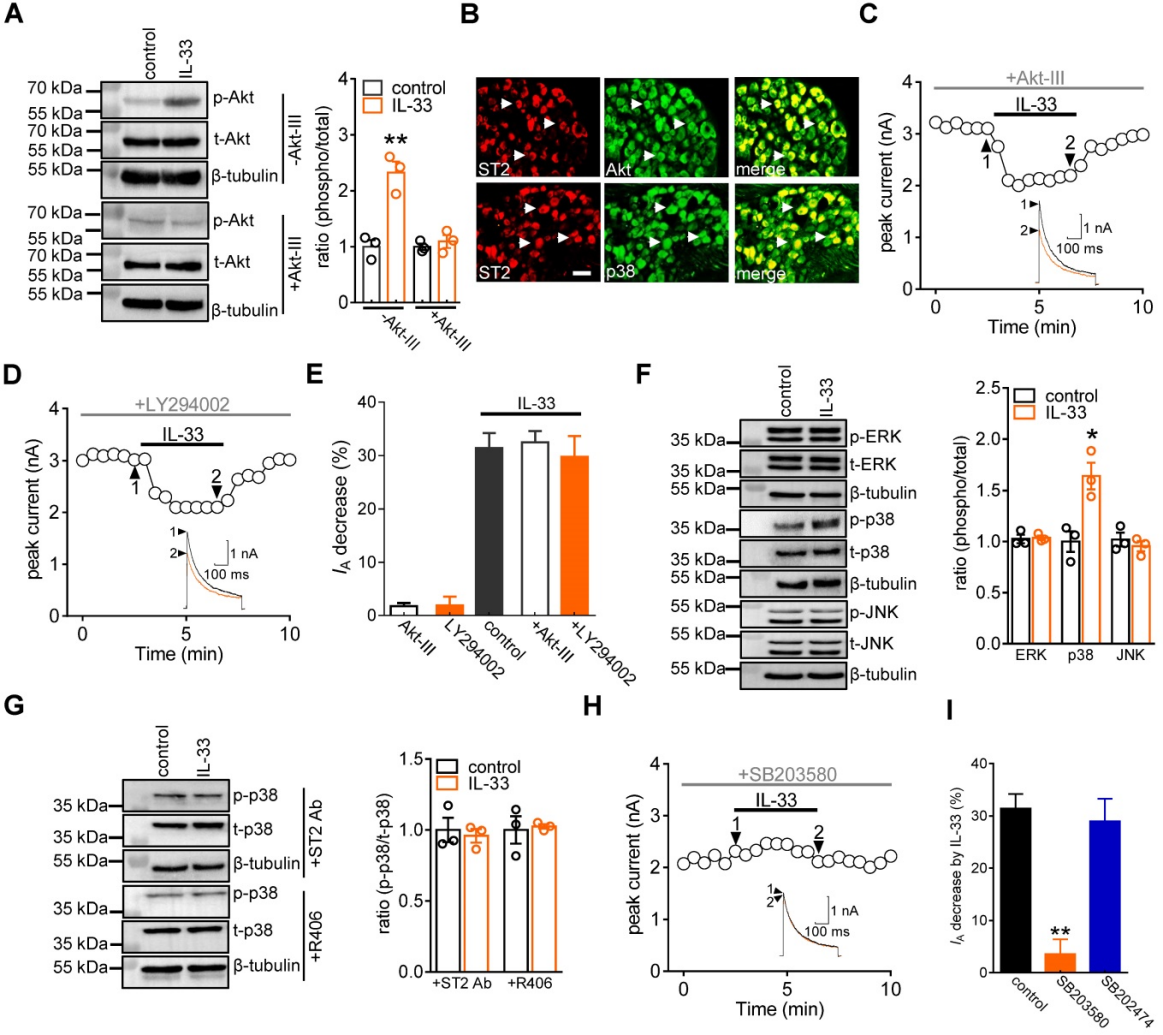
** IL-33 decreases *I*_A_ via p38 MAPK. A,** effects of 50 ng/mL IL-33 on phospho-Akt (*p*-Akt) or total Akt (*t*-Akt) protein abundance in the presence or absence of Akt inhibitor III (Akt-III, 10 µM) in DRG cells. Blots are representative of three independent experiments with β-tubulin serving as a loading control. **p < 0.01* vs.* control, unpaired t test. **B,** colabeling (white arrows) of ST2 and Akt and p38 in mouse DRG sections. Scale bar, 50 µm. **C-D,** time course of *I*_A_ changes indicating the effects of IL-33 on *I*_A_ in the presence of 10 µM Akt inhibitor III (*C*) or 20 µM LY294002 (*D*). Arabic numerals indicate the points utilized for the example current traces. **E,** bar graph showing the effects of 50 ng/mL IL-33 on *I*_A_ in the presence of Akt inhibitor III (*n* = 10 cells) or LY294002 (*n* = 8 cells) as indicated in Panels *B* and *C*, respectively. Application of 10 µM Akt inhibitor III (*n* = 6 cells) or 20 µM LY294002 (*n* = 6 cells) alone did not significantly affect *I*_A_. **F,** effects of 50 ng/mL IL-33 on *p*-p38, *p*-JNK and* p*-ERK protein abundance in DRG cells. Blots are representative of three independent experiments with β-tubulin serving as a loading control. *p < 0.05* vs.* control, unpaired t test. **G,** pretreatment of DRG cells with the ST2 neutralizing antibody (ST2 Ab, 2 µg/mL) or R406 (1 µM) abolished the 50 ng/mL IL-33-induced increase in *p*-p38 protein abundance. Blots are representative of three independent experiments with β-tubulin serving as a loading control. **H,** time course of *I*_A_ changes indicating the effect of 50 ng/mL on *I*_A_ in cells pretreated with 10 µM SB203580. **I,** bar graph showing that pretreating cells with SB203580 (*n* = 10 cells), but not its inactive analogue SB202474 (10 µM, *n* = 6 cells), prevented the IL-33-induced *I*_A_ decrease. **p < 0.01 *vs.* control, paired t test.

IL-33/ST2 signaling triggers PI3K/Akt, which can be a downstream effector of Syk 45. Immunoblot analysis of DRG cell lysates revealed the protein expression of Akt (Figure 4A & Figure S6), and immunofluorescence staining showed that Akt was coexpressed with ST2 in DRG neurons (Figure 4B). Therefore, we determined whether the effect of IL-33 was dependent on PI3K/Akt activity. Treatment with 50 ng/mL IL-33 markedly increased the expression level of phosphorylated Akt (*p*-Akt) in DRG cells, and this effect was abrogated by 10 μM Akt inhibitor III pretreatment (Figure 4A & Figure S6). Interestingly, dialysis of small DRG neurons with Akt inhibitor III (10 μM) did not affect the IL-33-induced *I*_A_ response (decrease of 32.5 ± 2.2%, Figure 4C-E). Akt is a common downstream effector of PI3K, but Akt-independent PI3K activation has also been demonstrated 46. Further pretreatment with the PI3K inhibitor LY294002 did not influence the ability of IL-33 to decrease *I*_A_ (decrease of 29.8 ± 3.8%, Figure 4D-E). These results exclude the involvement of PI3K/Akt signaling in the IL-33/ST2-mediated *I*_A_ response. Mitogen-activated protein kinases (MAPKs) play critical roles in regulating pain responses 47 and are important molecules involved in *I*_A_ regulation 48. We next examined whether MAPK molecules participated in the IL-33/ST2-induced *I*_A_ decrease in DRG neurons. Immunoblotting showed that exposure of DRG cells to 50 ng/mL IL-33 markedly increased the expression level of *p*-p38, while the total p38 (t-p38),* p*-ERK and* p*-JNK levels remained unaffected (Figure 4F & Figure S7). Immunostaining of intact mouse DRGs indicated that p38 was coexpressed with ST2 (Figure 4B). Pretreating DRG cells with either an ST2 neutralizing antibody (2 μg/mL) or the Syk inhibitor R406 (1 µM) eliminated the IL-33-induced increase in p-p38 protein abundance (Figure 4G & Figure S8). Furthermore, pretreating neurons with 10 µM SB203580, a selective p38 MAPK inhibitor, completely prevented the IL-33-induced *I*_A_ decrease (decrease of 3.6 ± 2.8%, Figure 4H-I), while 10 µM SB202474, the inactive structural analogue of SB203580, elicited no such effect (decrease of 28.9 ± 4.3%, Figure 4I).

### p38β mediates the IL-33-induced *I*_A_ response

**Figure 5:**
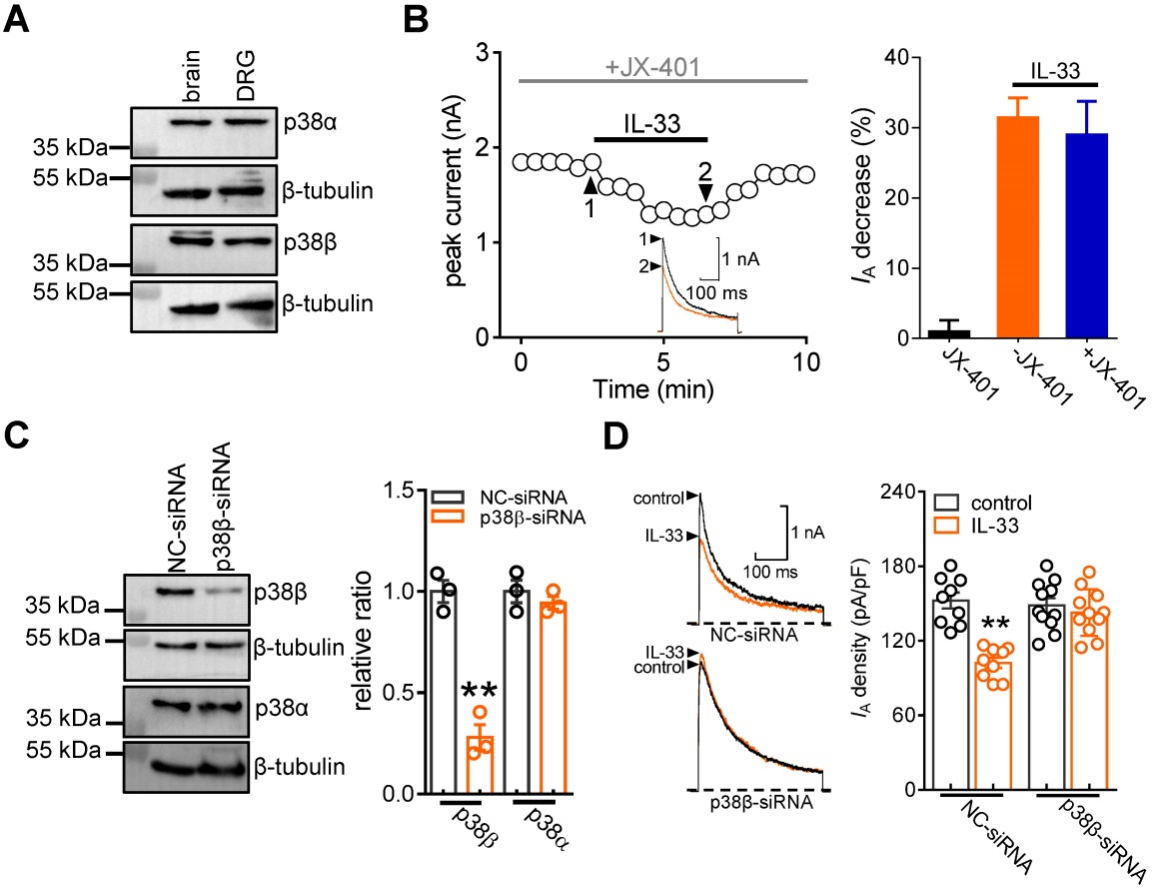
** p38β mediates the IL-33-induced *I*_A_ decrease. A,** immunoblot analysis of p38α and p38β protein abundance in DRGs. Mouse brains were used as positive controls. Blots are representative of three independent experiments with β-tubulin serving as a loading control. **B,** time course of *I*_A_ changes (*left*) and bar graph (*right*) indicating the effect of 50 ng/mL IL-33 on *I*_A_ in the presence of JX-401 (50 nM, *n* = 8 cells). The application of 50 nM JX-401 (*n* = 6 cells) alone had no significant effect on *I*_A_. Arabic numerals indicate the points utilized for the example current traces. **C,** immunoblot analysis showing that the protein expression level of p38β was significantly reduced in the p38β-siRNA-treated groups, while the expression of p38α was not affected. Blots are representative of three independent experiments with β-tubulin serving as a loading control. **p < 0.01 *vs.* NC-siRNA, unpaired t test. **D,** example traces (*left*) and bar graph (*right*) demonstrating the effects of 50 ng/mL IL-33 on *I*_A_ in cells treated with control siRNA (NC-siRNA, *n* = 9 cells) or p38β-siRNA (*n* = 11 cells). **p < 0.01 *vs.* control + NC-siRNA group, one-way ANOVA with a Bonferroni post hoc test.

The p38 MAPK family consists of four isoforms (p38α, β, δ, and γ), and only the α and β isoforms are expressed in the mature nervous system and involved in hyperalgesia 49-51. Thereafter, we determined the exact p38 isoform that participates in the IL-33-induced *I*_A_ response. Immunoblot analysis showed that both p38α and p38β were detected in adult mouse DRGs (Figure 5A & Figure S9). Preincubation of DRG neurons with the potent p38α inhibitor JX-401 (50 nM) did not affect the ability of IL-33 to decrease *I*_A_ (decrease 29.1 ± 4.8%, Figure 5B). We next determined the involvement of p38β-mediated signaling in the IL-33-mediated response. Due to the lack of a commercial chemical p38β inhibitor, we knocked down p38β expression using a siRNA-mediated approach in DRGs. Intrathecal administration of chemically modified p38β-siRNA resulted in a marked downregulation of p38β protein abundance in mouse DRGs, whereas the p38α expression level remained unaffected (Figure 5C & Figure S10). Knockdown of p38β completely abolished the 50 ng/mL IL-33-induced *I*_A_ decrease in small DRG neurons (decrease of 3.3 ± 1.9%, Figure 5D).

### IL-33/ST2 enhances DRG neuronal excitability

**Figure 6:**
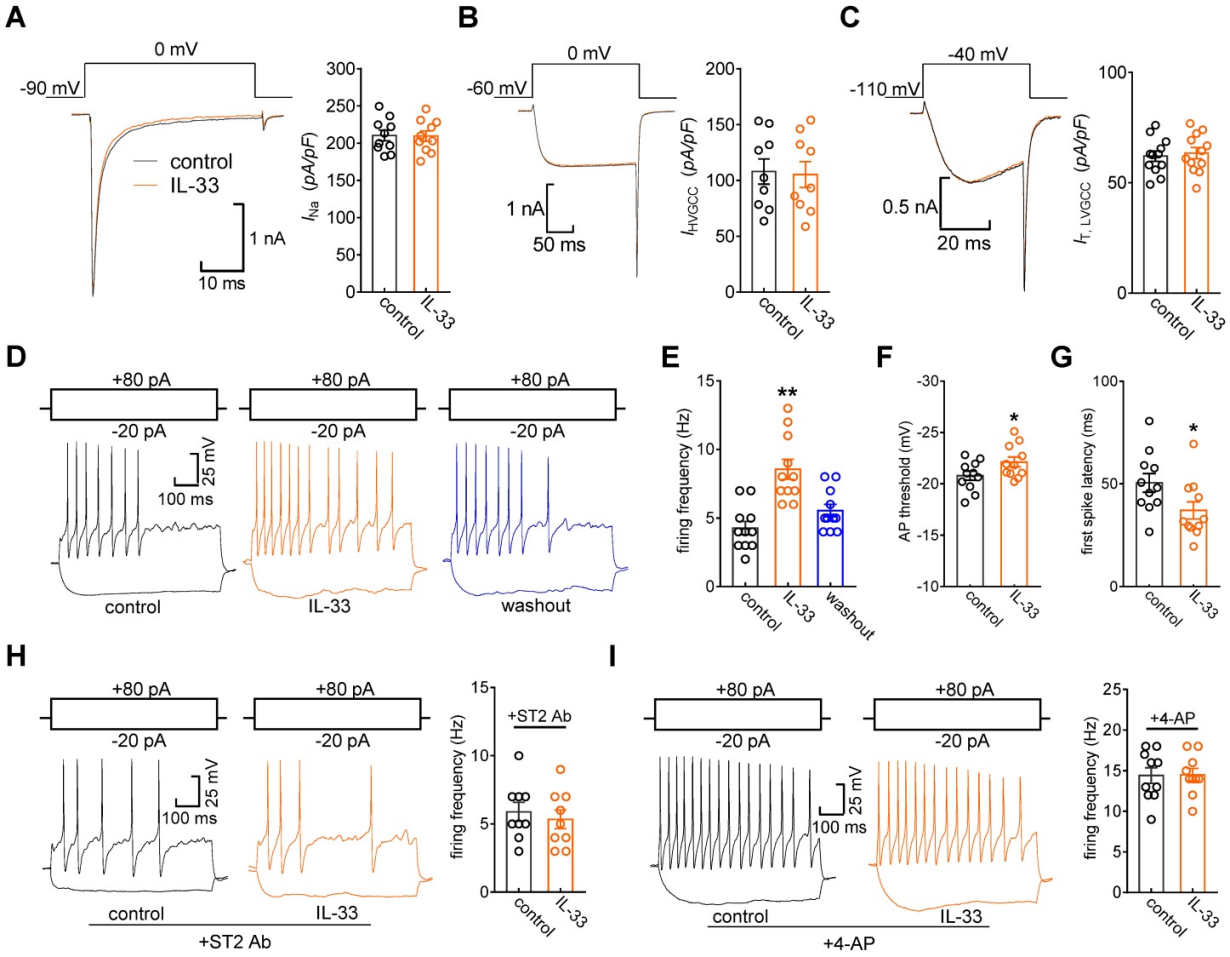
** IL-33 increases DRG neuronal excitability. A-C,** example traces (*left*) and summary data (*right*) demonstrating that 50 ng/mL IL-33 did not affect Nav currents (*I*_Na_, *A*, *n* = 10 cells), high voltage-activated (HVA) Cav currents (*I*_HVACC_, *B*, *n* = 9 cells) or low voltage-activated (LVA, T-type) Cav currents (*I*_T_,* C*, *n* = 12 cells). Nav currents were evoked by a test pulse to 0 mV from the holding potential of -90 mV. *I*_HVACC_s were elicited by a depolarizing step to 0 mV with a holding potential of -60 mV. To isolate *I*_T_, we blocked *I*_HVACC_ by bath-applying a cocktail of channel blockers, including 5 µM nifedipine, 0.2 µM ω-conotoxin MVIIC, and 0.2 µM SNX482. *I*_T_ was elicited by a 40-ms depolarizing step pulse from the holding potential (-110 mV) to the command voltage of -40 mV. **D-E,** example traces (*D*) and bar graph (*E*) demonstrating that 50 ng/mL IL-33 increased the rate of action potential (AP) firing (*n* = 11 cells). **p < 0.01 *vs.* control, paired t test. **F-G,** IL-33 (50 ng/mL) lowered the AP threshold (*F*) and shortened the first-spike latency (*G*) (*n* = 11 cells). *p < 0.05 *vs.* control, paired t test. **H-I,** example traces (*left*) and summary data (*right*) demonstrating that pretreatment with the ST2 neutralizing antibody (2 µg/mL, *H*, *n* = 9 cells) or 4-AP (5 mM, *I*, *n* = 10 cells) abrogated the increased AP firing rate induced by 50 ng/mL IL-33.

Kv channels play an essential role in modulating membrane excitability in many cell types, including sensory neurons in DRGs. To examine the functional roles of IL-33/ST2 in the regulation of *I*_A_, we tested whether IL-33 affects DRG neuronal excitability. The application of 50 ng/mL IL-33 to small neurons did not affect voltage-gated Na^+^ channel (Nav) currents (decrease of 0.8 ± 0.3%, Figure 6A). In addition, IL-33 at 50 ng/mL had no significant effects on high voltage-activated (HVA) Cav currents (decrease of 2.7 ± 1.6%, Figure 6B) or low voltage-activated (LVA, T-type) Cav currents (decrease of -2.2 ± 1.5%, Figure 6C). Bath application of IL-33 (50 ng/mL) markedly increased the rate of action potential (AP) firing (102.2 ± 9.6%, Figure 6D-E). In addition, the application of 50 ng/mL IL-33 lowered the AP threshold (Figure 6F) and shortened the first-spike latency (Figure 6G). Other membrane properties, including input resistance and resting membrane potential, were not affected (not shown). The IL-33-induced increase in the AP firing rate was completely prevented by pretreatment with the ST2 neutralizing antibody (2 μg/mL, Figure 6H). To further verify the neuronal hyperexcitability induced by IL-33/ST2 through *I*_A_ regulation, we added 5 mM 4-AP to the bath solution to block *I*_A_ and found that preincubation with 4-AP abrogated IL-33-induced neuronal hyperexcitability in DRG neurons (Figure 6I).

### Involvement of *I*_A_ in IL-33-induced pain hypersensitivity *in vivo*

**Figure 7:**
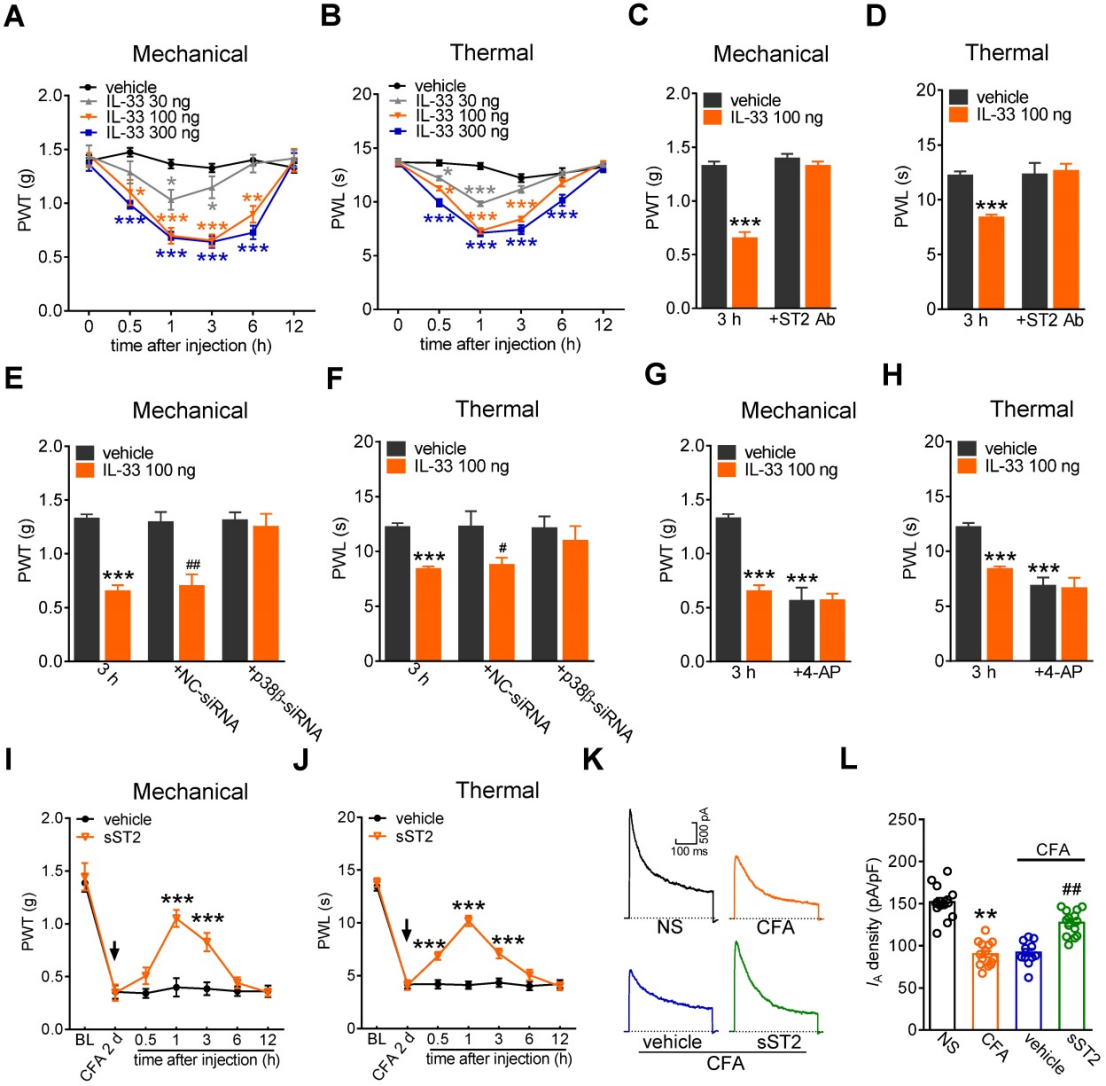
** IL-33/ST2 signaling participates in peripheral pain sensitivity. A-B,** intraplantar injection (i.p.l.) of IL-33 at 30 ng, 100 ng, and 300 ng significantly decreased the mechanical paw withdrawal threshold (PWT,* A*) and heat paw withdrawal latency (PWL,* B*). *p < 0.05, **p < 0.01, ***p < 0.001, *vs.* vehicle at the corresponding points, two-way ANOVA with a Bonferroni post hoc test. **C-D,** intraplantar pretreatment with 1 µg of ST2 neutralizing antibody (ST2 Ab) completely prevented the mechanical (*C*) and heat (*D*) hypersensitivity induced by 100 ng of IL-33 (i.p.l.). ***p < 0.001 *vs.* vehicle at 3 h, two-way ANOVA with a Bonferroni post hoc test. **E-F,** p38β siRNA attenuated mechanical and heat hypersensitivity induced by 100 ng of IL-33 (i.p.l.). ***p < 0.001 *vs.* vehicle at 3 h, ^#^*P* < 0.05, ^##^*P* < 0.01 *vs.* vehicle in the NC-siRNA groups, two-way ANOVA with a Bonferroni post hoc test. **G-H,** intraplantar pretreatment with 25 nmol 4-AP occluded mechanical (*G*) and heat (*H*) hypersensitivity mediated by 100 ng of IL-33. ***p < 0.001 *vs.* vehicle at 3 h, two-way ANOVA with a Bonferroni post hoc test. **I-J,** intraplantar injection of sST2 at 2 µg attenuated the mechanical hypersensitivity (*I*) and thermal hyperalgesia (*J*) in CFA mice. The arrow indicates the injection of sST2 or vehicle. ***p < 0.001 *vs.* vehicle at the corresponding points, two-way ANOVA with a Bonferroni post hoc test. **K-L,** representative current traces (*K*) and summary data (*L*) indicating that intraplantar injection of sST2 (2 µg) abolished the CFA (2 d)-induced *I*_A_ decrease in small-sized DRG neurons (*n* = 11-14 neurons per group). **p < 0.01 compared with the normal saline (NS) group, ##*p* < 0.01 compared with the CFA + vehicle group, one-way ANOVA with a Bonferroni post hoc test. *N* = at least 7 mice for all animal behavior experiments.

Furthermore, to examine the contribution of IL-33/ST2 signaling to behavioral signs of pain, we determined whether peripheral application of IL-33 affected pain sensitivity in mice. Intraplantar injection of 30 ng, 100 ng, and 300 ng of IL-33 induced significant decreases in the paw withdrawal thresholds (PWT, Figure 7A) and paw withdrawal latencies (PWL, Figure 7B), indicating a significant increase in sensitivity to both acute mechanical and heat stimuli. IL-33 at 100 ng and 300 ng showed stronger effects than those after treatment with 30 ng. These effects started at 30 min, were maintained for more than 3 h and recovered after 6 h (Figure 7A-B). The 100 ng IL-33-induced mechanical and heat hypersensitivity was abolished by prior intraplantar injection with 1 µg of the ST2 neutralizing antibody (Figure 7C-D). Next, we determined whether p38β participated in IL-33-induced pain hypersensitivity. Lumbar intrathecal injection of siRNA led to preferential uptake in the corresponding DRGs (Figure S11), which is consistent with previous studies 52, 53. Compared with the control siRNA-treated groups, intrathecal administration of p38β siRNA resulted in a significant decrease in p38β protein abundance in lumbar DRGs (Figure 5C). Intrathecal delivery of p38β siRNA prevented the mechanical and heat hypersensitivity induced by 100 ng of IL-33, while delivery of the control siRNA did not elicit such effects in either nociceptive behavioral test (Figure 7E-F). Moreover, intraplantar pretreatment with 4-AP (25 nmol) resulted in a significant hypersensitivity to acute mechanical stimulus and heat (Figure 7G-H). Assessment of the sensitivity after IL-33 injection (100 ng) showed that IL-33 had no significant additive effect on mechanical (Figure 7G) or heat (Figure 7H) pain sensitivity. These results together suggest that *I*_A_ channels contribute to ST2-mediated hypersensitivity to acute pain. Furthermore, the endogenous role of IL-33 in chronic inflammatory pain was determined. Mice exhibited significant mechanical hypersensitivity and thermal hyperalgesia to complete Freund's adjuvant (CFA; Figure 7I-J). Intraplantar injection of 2 µg of the soluble form of ST2 (sST2), a decoy receptor that neutralizes IL‑33, attenuated CFA-induced mechanical hypersensitivity (Figure 7I) and thermal hyperalgesia (Figure 7J), with the effect sustained for 3 hours. Moreover, CFA significantly decreased the current density of the *I*_A_ in small-sized DRG neurons (Figure 7K-L), and this effect was abolished by intraplantar injection of sST2 (Figure 7K-L). These results together demonstrate that the IL-33-induced nociceptive behaviors is mediated by ST2 and *I*_A_ channels modulation.

## Discussion

**Figure 8:**
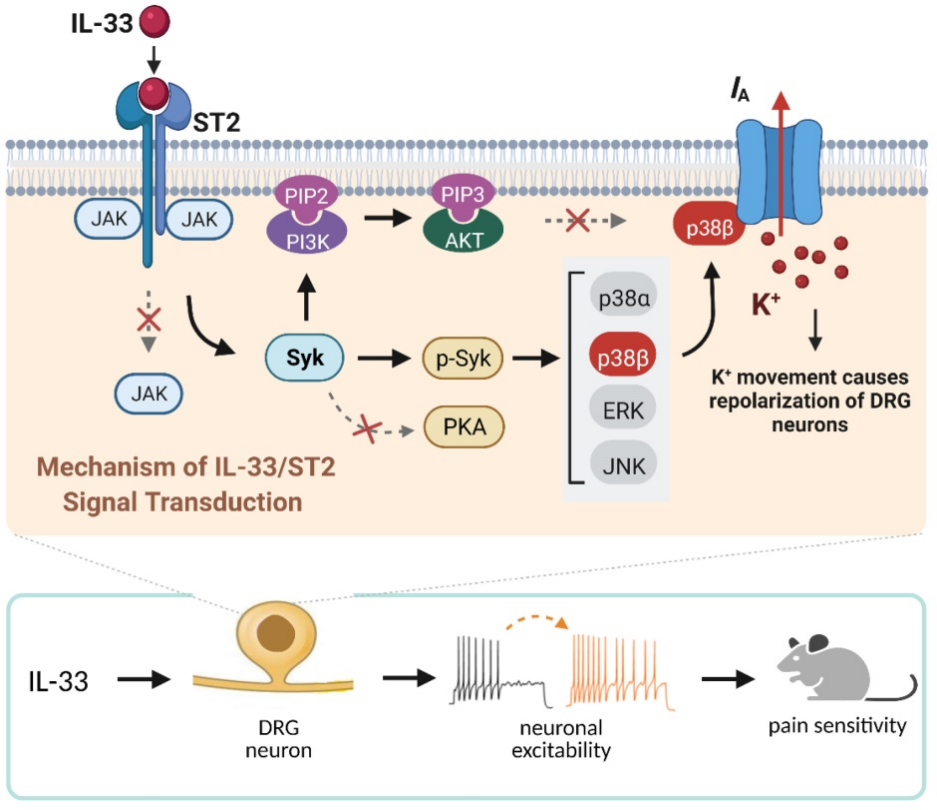
** Schematic showing the molecular mechanism of IL-33-induced neuronal hyperexcitability of DRG neurons and pain hypersensitivity in mice.** IL-33 acting through ST2 receptors does not affect the activity of JAK2 but leads to the activation of Syk. The increased level of *p*-Syk stimulates downstream p38β signaling, which in turn regulates A-type channel activity and results in *I*_A_ reduction. IL-33/ST2-mediated signaling enhances neuronal excitability of DRG neurons and nociceptive behaviors in mice. Neither PKA nor PI3K/Akt was necessary for the IL-33-induced *I*_A_ response in this study. Whether p38β directly phosphorylates the channels encoding *I*_A_ or stimulates intermediate molecules in small DRG neurons needs to be investigated further. Created with BioRender.com.

The present study elucidates the detailed signaling pathway that initiated by IL-33/ST2 in modulating *I*_A_ in peripheral sensory neurons. We revealed that IL-33 suppressed *I*_A_ via its membrane ST2, which was coupled to JAK2-independent and Syk-dependent p38β signaling (see Figure 8). This IL-33/ST2-mediated *I*_A_ suppression induced sensory neuronal hyperexcitability in DRG neurons and pain hypersensitivity in mice.

Immunostaining of ST2 on DRG has demonstrated that ST2 is mainly expressed in CGRP-labelled peptidergic neurons and IB_4_-labelled nonpeptidergic neurons, but to a lesser extent in neurofilament-200 (NF200)-labelled neurons. Previous studies have also shown that ST2 is expressed directly on myelinated (NF200-positive) and non-myelinated (CGRP- and IB4-positive) sensory neurons 13, suggesting a direct action of IL-33 on mouse DRG neurons, which is consistent with the present study. It has been demonstrated that IL-33 mediates mast cell activation by targeting the PI3K/Akt axis 54. Additionally, in the pancreas, IL-33 increases the expression of phosphorylated Akt and phosphorylated PI3K 55. Interestingly, studies examining the PI3K/Akt-dependent modulation of Kv4, which forms one of the major components mediating I_A_, have led to conflicting conclusions**.** In rat pancreatic β cells, Kv currents, including *I*_A_, decrease in response to PI3K/Akt activation 56. Similarly, in cultured embryonic mouse hippocampal neurons, PI3K/Akt signaling mediated the inhibition of *I*_A_ induced by astroglial contact 57. In contrast, stimulation of PI3K/Akt has been shown to increase *I*_A_ in rat cerebellar granule cells 58. A PI3K-induced increase in Kv4.3 channel current through glucocorticoid inducible kinase-1 has also been identified 59. Interestingly, in peripheral sensory neurons, Akt-independent PI3K was shown to regulate transient outward* I*_A_ 60. Thus, Akt acts to differentially regulate Kv4 channel activity in a tissue-/cell type-specific manner. In this study, IL-33/ST2-mediated *I*_A_ inhibition in DRG neurons was identified to be PI3K/Akt-independent, since IL-33/ST2 neither increased the level of p-Akt nor antagonized PI3K/Akt signaling to affect the IL-33-induced *I*_A_ response. It has been well established by* in vivo* and *in vitro* studies that ERK-mediated signaling plays a critical role in nociceptive pain behaviors 47, 61. Peripheral nerve injury induced elevated levels of p-ERK in DRG cells 62. ERK was identified to directly phosphorylate the pore-forming α subunit of Kv4.2 63, and stimulation of ERK resulted in a downregulation of *I*_A_ in superficial spinal dorsal horn neurons 48. In contrast, in lateral pyloric neurons, ERK signaling has been shown to mediate the *I*_A_ increase induced by dopamine 64, while the inhibition of MEK by PD98059 displayed no significant effect in CA1 pyramidal neurons 65. In our present study, stimulation with ST2 elevated the level of *p*-p38 in mouse DRG cells, whereas *p*-ERK and *p*-JNK remained unaffected. These findings exclude the possibility of ERK involvement in the IL-33/ST2-mediated *I*_A_ decrease. Moreover, further investigations demonstrated that the suppression of *I*_A_ induced by IL-33 was not affected by the MAPK/ERK inhibitor. The present findings identified that Syk stimulated downstream p38β and that this signaling in DRG neurons is required for the IL-33/ST2 effects. Our data indicated that 1) the p38 MAPK inhibitor abrogated the IL-33-induced *I*_A_ reduction; 2) Syk antagonism prevented IL-33/ST2-mediated p38 activation; and 3) siRNA knockdown of p38β completely abolished the *I*_A_ decrease induced by IL-33. These findings are consistent with previous studies in rat ventricular myocytes showing that activation of p38 MAPK resulted in a reduction in transient outward K^+^ currents 66. Further observations in hippocampal pyramidal neurons showed that the increased activity of p38 markedly decreased Kv currents by phosphorylation of Kv4.2 channels 65, 67. In contrast, it has been demonstrated that p38 stimulates Kv currents in transfected Chinese hamster ovary cells 68. Further investigations also revealed the involvement of p38 MAPK in the enhancement of Kv1.3 currents 69. Although these discrepancies need to be investigated further, the regulatory effects of different p38 subtypes would vary in cell types/tissues expressing distinct channel types encoding *I*_A_. An alternative hypothesis is that p38β in DRG neurons can also phosphorylate an intermediate protein, resulting in the downregulation of *I*_A_. In addition, different splice variants of K^+^ channel-interacting protein would engender different, even opposing, regulation of Kv4 currents 70.

Painful conditions such as hyperalgesia and allodynia *in vivo* can be directly affected by changes in peripheral neural excitability 17, 18. As the key components regulating membrane excitability, *I*_A_ channels have been implicated in the control of both spike frequency and first-spike latency 22, 71, the two pivotal determinants of the processes underlying neurotransmitter release, and hence nociceptive processing 72. An important outcome of peripheral *I*_A_ modulation is the influence of somatic and visceral nociceptive inputs, and *I*_A_ reduction has been shown to induce significant nociception in a variety of neuropathic pain models 38. Consistent with the* I*_A_ decrease induced by IL-33, in this study, stimulation with ST2 markedly increased DRG neuronal excitability along with an increased AP firing rate and shorter first-spike latency. Previously, IL-33 has been shown to induce significant calcium influx following activation of the innate immune system in sensory neurons 13, indicative of increased excitability. In addition, administration of an ST2 neutralizing antibody abolished the IL-33-induced increase in [Ca^2+^]_i_, functionally validated a direct role of ST2 in IL-33-induced neuronal hyperexcitability. Moreover, inhibition of* I*_A_ occludes IL-33-induced acute pain hypersensitivity. As such, although potential channel targets such as TRP-like channels can also be activated by ST2-mediated signaling 73, it is reasonable to infer that the nociceptive effects of IL-33/ST2 are mediated, in whole or in part, via *I*_A_ suppression. Indeed, our current findings are aligned with prior *in vivo* studies showing that intrathecal injection of either IL-33 or an ST2 neutralizing antibody alleviated mechanical allodynia 13, 14 in rodents and that activation of ST2 caused hyperalgesia in various pain models 8, 40, 74. Moreover, genetically deleting IL-33 and its receptor ST2 significantly ameliorated pain hypersensitivity in a mouse gout model 15. In line with our present findings, Huang *et al.* reported that the intraplantar injection of IL-33 resulted in increased sensitivity to thermal and mechanical stimuli. Intriguingly, ST2 receptors were found to be expressed in the superficial skin tissue of the hindpaw, albeit not on sensory nerve endings 13. This suggests that another signaling molecule is required to sensitize the sensory neurons in the paw. On the other hand, the ST2 receptors were found on the cell bodies of DRGs and intrathecally injected IL-33 caused sensitization by a direct action on ST2 receptors on neurons. In particular, Huang *et al.* suggest a two-step process of peripheral sensitization/priming of sensory neurons that then manifested itself at the level of the DRG, and this fits quite nicely with our present study that intrathecal injection of ST2 receptor antibodies blocked the effects of intraplantar IL-33. Although further investigation is necessary, it seems that there are two sites of action of IL-33 in regulating nociceptive responses-in the paw and at the level of the DRG. Nevertheless, previous studies have reported that the distinct roles of p38α and p38β in pain are model-dependent 50, 51, 75. Svensson *et al.* demonstrated that spinal p38β, but not the p38α, mediates tissue injury-induced hyperalgesia 50. Consistently, in this study, intrathecal administration of chemically modified p38β-siRNA resulted in a marked downregulation of p38β protein abundance in DRGs, and prevented the pain hypersensitivity induced by intraplantar injection of IL-33. It should be noted that lumbar intrathecal injection of chemically modified siRNA led to preferential uptake in the corresponding DRGs, which is consistent with previous studies 52, 53. In contrast, intrathecal application of antisense oligonucleotides against p38α but not the p38β attenuates neuropathic and postoperative pain 51. Interestingly, both p38α and p38β have been reported to be involved in the generation and maintenance of bone cancer pain states 75. Although these discrepancies have yet to be explained, these observations suggest that different modes of pain sensitivity and transmission may not necessarily be stimulated by similar pathways involving different p38 MAPK isoforms. In addition, distinct expression patterns of p38α and p38β may also contribute. For instance, in the spinal dorsal horn, the isoforms of p38 are distinctly expressed: p38α is expressed in neurons and p38β in microglia 50, 76, while in peripheral sensory neurons, both p38α and p38β MAPK are endogenously expressed 51.

Taken together, we identified the novel molecular circuit underlying the IL-33-mediated reduction in *I*_A_ in small DRG neurons in mice, whereas the *I*_DR_ remained unaffected. Our findings revealed that JAK2-independent ST2 stimulation and downstream Syk-mediated p38β signaling contribute to IL-33-driven sensory neuronal hyperexcitability and are associated with pain hypersensitivity *in vivo*. The knowledge of IL-33/ST2-mediated signaling gained in the present study may pave the way for p38β to be developed as a potential therapeutic target for the clinical management of pain.

## Supplementary Material

Supplementary figures.

## Abbreviations

IL-33: interleukin-33
ST2 receptor: suppression of tumorigenicity 2 (ST2) receptor
DRG: dorsal root ganglion
Kv: voltage-gated K^+^ channels
*I*_A_: transient outward K^+^ channel currents
4-AP: 4-aminopyridine
MAPK: mitogen-activated protein kinase
ERK: extracellular regulated protein kinases
PKA: protein kinase A

## References

[1] Fairlie-Clarke K, Barbour M, Wilson C, Hridi SU, Allan D, Jiang HR (2018). Expression and Function of IL-33/ST2 Axis in the Central Nervous System Under Normal and Diseased Conditions. Front Immunol.

[2] Palmer G, Gabay C (2011). Interleukin-33 biology with potential insights into human diseases. Nat Rev Rheumatol.

[3] Sanada S, Hakuno D, Higgins LJ, Schreiter ER, McKenzie AN, Lee RT (2007). IL-33 and ST2 comprise a critical biomechanically induced and cardioprotective signaling system. J Clin Invest.

[4] Liew FY, Girard JP, Turnquist HR (2016). Interleukin-33 in health and disease. Nat Rev Immunol.

[5] Chen Z, Hu Q, Huo Y, Zhang R, Fu Q, Qin X (2021). Serum Interleukin-33 is a Novel Predictive Biomarker of Hemorrhage Transformation and Outcome in Acute Ischemic Stroke. J Stroke Cerebrovasc Dis.

[6] Du LX, Wang YQ, Hua GQ, Mi WL (2018). IL-33/ST2 Pathway as a Rational Therapeutic Target for CNS Diseases. Neuroscience.

[7] Sacmaci H, Sabah Ozcan S (2020). A critical role for expression of atypical chemokine receptor 2 in multiple sclerosis: A preliminary project. Mult Scler Relat Disord.

[8] Alvarez P, Bogen O, Levine JD (2020). Nociceptor Interleukin 33 Receptor/ST2 Signaling in Vibration-Induced Muscle Pain in the Rat. J Pain.

[9] Yin C, Liu B, Wang P, Li X, Li Y, Zheng X (2020). Eucalyptol alleviates inflammation and pain responses in a mouse model of gout arthritis. Br J Pharmacol.

[10] Fattori V, Hohmann MSN, Rossaneis AC, Manchope MF, Alves-Filho JC, Cunha TM (2017). Targeting IL-33/ST2 signaling: regulation of immune function and analgesia. Expert Opin Ther Targets.

[11] Verri WA Jr, Guerrero AT, Fukada SY, Valerio DA, Cunha TM, Xu D (2008). IL-33 mediates antigen-induced cutaneous and articular hypernociception in mice. Proc Natl Acad Sci U S A.

[12] Zarpelon AC, Cunha TM, Alves-Filho JC, Pinto LG, Ferreira SH, McInnes IB (2013). IL-33/ST2 signalling contributes to carrageenin-induced innate inflammation and inflammatory pain: role of cytokines, endothelin-1 and prostaglandin E2. Br J Pharmacol.

[13] Huang J, Gandini MA, Chen L, M'Dahoma S, Stemkowski PL, Chung H (2020). Hyperactivity of Innate Immunity Triggers Pain via TLR2-IL-33-Mediated Neuroimmune Crosstalk. Cell Rep.

[14] Zeng Y, Shi Y, Zhan H, Liu W, Cai G, Zhong H (2020). Reduction of Silent Information Regulator 1 Activates Interleukin-33/ST2 Signaling and Contributes to Neuropathic Pain Induced by Spared Nerve Injury in Rats. Front Mol Neurosci.

[15] Yin C, Liu B, Li Y, Li X, Wang J, Chen R (2020). IL-33/ST2 induces neutrophil-dependent reactive oxygen species production and mediates gout pain. Theranostics.

[16] Duan L, Huang Y, Su Q, Lin Q, Liu W, Luo J (2016). Potential of IL-33 for Preventing the Kidney Injury via Regulating the Lipid Metabolism in Gout Patients. J Diabetes Res.

[17] Julius D, Basbaum AI (2001). Molecular mechanisms of nociception. Nature.

[18] Waxman SG, Zamponi GW (2014). Regulating excitability of peripheral afferents: emerging ion channel targets. Nat Neurosci.

[19] Cai SQ, Li W, Sesti F (2007). Multiple modes of a-type potassium current regulation. Curr Pharm Des.

[20] Rasband MN, Park EW, Vanderah TW, Lai J, Porreca F, Trimmer JS (2001). Distinct potassium channels on pain-sensing neurons. Proc Natl Acad Sci U S A.

[21] Zemel BM, Ritter DM, Covarrubias M, Muqeem T (2018). A-Type KV Channels in Dorsal Root Ganglion Neurons: Diversity, Function, and Dysfunction. Front Mol Neurosci.

[22] Hu HJ, Carrasquillo Y, Karim F, Jung WE, Nerbonne JM, Schwarz TL (2006). The kv4.2 potassium channel subunit is required for pain plasticity. Neuron.

[23] Duan KZ, Xu Q, Zhang XM, Zhao ZQ, Mei YA, Zhang YQ (2012). Targeting A-type K(+) channels in primary sensory neurons for bone cancer pain in a rat model. Pain.

[24] Carrasquillo Y, Nerbonne JM (2014). IA channels: diverse regulatory mechanisms. Neuroscientist.

[25] Chien LY, Cheng JK, Chu D, Cheng CF, Tsaur ML (2007). Reduced expression of A-type potassium channels in primary sensory neurons induces mechanical hypersensitivity. J Neurosci.

[26] Takeda M, Tsuboi Y, Kitagawa J, Nakagawa K, Iwata K, Matsumoto S (2011). Potassium channels as a potential therapeutic target for trigeminal neuropathic and inflammatory pain. Mol Pain.

[27] Zhang Y, Qin W, Qian Z, Liu X, Wang H, Gong S (2014). Peripheral pain is enhanced by insulin-like growth factor 1 through a G protein-mediated stimulation of T-type calcium channels. Sci Signal.

[28] Zhang Y, Jiang D, Zhang Y, Jiang X, Wang F, Tao J (2012). Neuromedin U type 1 receptor stimulation of A-type K+ current requires the betagamma subunits of Go protein, protein kinase A, and extracellular signal-regulated kinase 1/2 (ERK1/2) in sensory neurons. J Biol Chem.

[29] Guo Q, Jiang YJ, Jin H, Jiang XH, Gu B, Zhang YM (2013). Modulation of A-type K+ channels by the short-chain cobrotoxin through the protein kinase C-delta isoform decreases membrane excitability in dorsal root ganglion neurons. Biochem Pharmacol.

[30] Andre S, Boukhaddaoui H, Campo B, Al-Jumaily M, Mayeux V, Greuet D (2003). Axotomy-induced expression of calcium-activated chloride current in subpopulations of mouse dorsal root ganglion neurons. J Neurophysiol.

[31] Zhang Y, Zhang Y, Wang Z, Sun Y, Jiang X, Xue M (2021). Suppression of delayed rectifier K(+) channels by gentamicin induces membrane hyperexcitability through JNK and PKA signaling pathways in vestibular ganglion neurons. Biomed Pharmacother.

[32] Cao J, Zhang Y, Wu L, Shan L, Sun Y, Jiang X (2019). Electrical stimulation of the superior sagittal sinus suppresses A-type K(+) currents and increases P/Q- and T-type Ca(2+) currents in rat trigeminal ganglion neurons. J Headache Pain.

[33] Wang F, Zhang Y, Jiang X, Zhang Y, Zhang L, Gong S (2011). Neuromedin U inhibits T-type Ca2+ channel currents and decreases membrane excitability in small dorsal root ganglia neurons in mice. Cell Calcium.

[34] Wang H, Wei Y, Pu Y, Jiang D, Jiang X, Zhang Y (2019). Brain-derived neurotrophic factor stimulation of T-type Ca(2+) channels in sensory neurons contributes to increased peripheral pain sensitivity. Sci Signal.

[35] Dixon WJ (1980). Efficient analysis of experimental observations. Annu Rev Pharmacol Toxicol.

[36] Kawasaki Y, Xu ZZ, Wang X, Park JY, Zhuang ZY, Tan PH (2008). Distinct roles of matrix metalloproteases in the early- and late-phase development of neuropathic pain. Nat Med.

[37] Tan PH, Yang LC, Shih HC, Lan KC, Cheng JT (2005). Gene knockdown with intrathecal siRNA of NMDA receptor NR2B subunit reduces formalin-induced nociception in the rat. Gene Ther.

[38] Duan KZ, Xu Q, Zhang XM, Zhao ZQ, Mei YA, Zhang YQ (2012). Targeting A-type K(+) channels in primary sensory neurons for bone cancer pain in a rat model. Pain.

[39] Winkelman DL, Beck CL, Ypey DL, O'Leary ME (2005). Inhibition of the A-type K+ channels of dorsal root ganglion neurons by the long-duration anesthetic butamben. J Pharmacol Exp Ther.

[40] Liu S, Mi WL, Li Q, Zhang MT, Han P, Hu S (2015). Spinal IL-33/ST2 Signaling Contributes to Neuropathic Pain via Neuronal CaMKII-CREB and Astroglial JAK2-STAT3 Cascades in Mice. Anesthesiology.

[41] Funakoshi-Tago M, Tago K, Sato Y, Tominaga S, Kasahara T (2011). JAK2 is an important signal transducer in IL-33-induced NF-κB activation. Cell Signal.

[42] Mun SH, Ko NY, Kim HS, Kim JW, Kim DK, Kim AR (2010). Interleukin-33 stimulates formation of functional osteoclasts from human CD14(+) monocytes. Cell Mol Life Sci.

[43] Pellefigues C, Mehta P, Chappell S, Yumnam B, Old S, Camberis M (2021). Diverse innate stimuli activate basophils through pathways involving Syk and IκB kinases. Proc Natl Acad Sci U S A.

[44] Yu S, Huang H, Iliuk A, Wang WH, Jayasundera KB, Tao WA (2013). Syk inhibits the activity of protein kinase A by phosphorylating tyrosine 330 of the catalytic subunit. J Biol Chem.

[45] Sumaria N, Martin S, Pennington DJ (2021). Constrained TCRgammadelta-associated Syk activity engages PI3K to facilitate thymic development of IL-17A-secreting gammadelta T cells. Sci Signal.

[46] Zhang Y, Ying J, Jiang D, Chang Z, Li H, Zhang G (2015). Urotensin-II receptor stimulation of cardiac L-type Ca2+ channels requires the betagamma subunits of Gi/o-protein and phosphatidylinositol 3-kinase-dependent protein kinase C beta1 isoform. J Biol Chem.

[47] Ji RR, Gereau RWt, Malcangio M, Strichartz GR (2009). MAP kinase and pain. Brain Res Rev.

[48] Hu HJ, Glauner KS, Gereau RWt (2003). ERK integrates PKA and PKC signaling in superficial dorsal horn neurons. I. Modulation of A-type K+ currents. J Neurophysiol.

[49] Ji RR, Suter MR (2007). p38 MAPK, microglial signaling, and neuropathic pain. Mol Pain.

[50] Svensson CI, Fitzsimmons B, Azizi S, Powell HC, Hua XY, Yaksh TL (2005). Spinal p38beta isoform mediates tissue injury-induced hyperalgesia and spinal sensitization. J Neurochem.

[51] Luo X, Fitzsimmons B, Mohan A, Zhang L, Terrando N, Kordasiewicz H (2018). Intrathecal administration of antisense oligonucleotide against p38alpha but not p38beta MAP kinase isoform reduces neuropathic and postoperative pain and TLR4-induced pain in male mice. Brain Behav Immun.

[52] Messinger RB, Naik AK, Jagodic MM, Nelson MT, Lee WY, Choe WJ (2009). *In vivo* silencing of the Ca(V)3.2 T-type calcium channels in sensory neurons alleviates hyperalgesia in rats with streptozocin-induced diabetic neuropathy. Pain.

[53] Bourinet E, Alloui A, Monteil A, Barrere C, Couette B, Poirot O (2005). Silencing of the Cav3.2 T-type calcium channel gene in sensory neurons demonstrates its major role in nociception. EMBO J.

[54] Nakajima S, Ishimaru K, Kobayashi A, Yu G, Nakamura Y, Oh-Oka K (2019). Resveratrol inhibits IL-33-mediated mast cell activation by targeting the MK2/3-PI3K/Akt axis. Sci Rep.

[55] Lu J, Liang Y, Zhao J, Meng H, Zhang X (2019). Interleukin-33 prevents the development of autoimmune diabetes in NOD mice. Int Immunopharmacol.

[56] El-Kholy W, Macdonald PE, Lin JH, Wang J, Fox JM, Light PE (2003). The phosphatidylinositol 3-kinase inhibitor LY294002 potently blocks K(V) currents via a direct mechanism. FASEB J.

[57] Wu RL, Butler DM, Barish ME (1998). Potassium current development and its linkage to membrane expansion during growth of cultured embryonic mouse hippocampal neurons: sensitivity to inhibitors of phosphatidylinositol 3-kinase and other protein kinases. J Neurosci.

[58] Yao JJ, Gao XF, Chow CW, Zhan XQ, Hu CL, Mei YA (2012). Neuritin activates insulin receptor pathway to up-regulate Kv4.2-mediated transient outward K+ current in rat cerebellar granule neurons. J Biol Chem.

[59] Lang F, Shumilina E (2013). Regulation of ion channels by the serum- and glucocorticoid-inducible kinase SGK1. FASEB J.

[60] Wang H, Qin J, Gong S, Feng B, Zhang Y, Tao J (2014). Insulin-like growth factor-1 receptor-mediated inhibition of A-type K(+) current induces sensory neuronal hyperexcitability through the phosphatidylinositol 3-kinase and extracellular signal-regulated kinase 1/2 pathways, independently of Akt. Endocrinology.

[61] Ma W, Quirion R (2005). The ERK/MAPK pathway, as a target for the treatment of neuropathic pain. Expert Opin Ther Targets.

[62] Sanna MD, Stark H, Lucarini L, Ghelardini C, Masini E, Galeotti N (2015). Histamine H4 receptor activation alleviates neuropathic pain through differential regulation of ERK, JNK and P38 MAPK phosphorylation. Pain.

[63] Schrader LA, Birnbaum SG, Nadin BM, Ren Y, Bui D, Anderson AE (2006). ERK/MAPK regulates the Kv4.2 potassium channel by direct phosphorylation of the pore-forming subunit. Am J Physiol Cell Physiol.

[64] Rodgers EW, Krenz WD, Jiang X, Li L, Baro DJ (2013). Dopaminergic tone regulates transient potassium current maximal conductance through a translational mechanism requiring D1Rs, cAMP/PKA, Erk and mTOR. BMC Neurosci.

[65] Hu JH, Malloy C, Tabor GT, Gutzmann JJ, Liu Y, Abebe D (2020). Activity-dependent isomerization of Kv4.2 by Pin1 regulates cognitive flexibility. Nat Commun.

[66] Li X, Li S, Xu Z, Lou MF, Anding P, Liu D (2006). Redox control of K+ channel remodeling in rat ventricle. J Mol Cell Cardiol.

[67] Hu JH, Malloy C, Hoffman DA (2020). P38 Regulates Kainic Acid-Induced Seizure and Neuronal Firing via Kv4.2 Phosphorylation. Int J Mol Sci.

[68] Redman PT, Hartnett KA, Aras MA, Levitan ES, Aizenman E (2009). Regulation of apoptotic potassium currents by coordinated zinc-dependent signalling. J Physiol.

[69] Liu J, Xu C, Chen L, Xu P, Xiong H (2012). Involvement of Kv1.3 and p38 MAPK signaling in HIV-1 glycoprotein 120-induced microglia neurotoxicity. Cell Death Dis.

[70] Van Hoorick D, Raes A, Keysers W, Mayeur E, Snyders DJ (2003). Differential modulation of Kv4 kinetics by KCHIP1 splice variants. Mol Cell Neurosci.

[71] Vydyanathan A, Wu ZZ, Chen SR, Pan HL (2005). A-type voltage-gated K+ currents influence firing properties of isolectin B4-positive but not isolectin B4-negative primary sensory neurons. J Neurophysiol.

[72] Tolhurst DJ, Smyth D, Thompson ID (2009). The sparseness of neuronal responses in ferret primary visual cortex. J Neurosci.

[73] Liu B, Tai Y, Achanta S, Kaelberer MM, Caceres AI, Shao X (2016). IL-33/ST2 signaling excites sensory neurons and mediates itch response in a mouse model of poison ivy contact allergy. Proc Natl Acad Sci U S A.

[74] Zhao J, Zhang H, Liu SB, Han P, Hu S, Li Q (2013). Spinal interleukin-33 and its receptor ST2 contribute to bone cancer-induced pain in mice. Neuroscience.

[75] Dong H, Tian YK, Xiang HB, Tian XB, Jin XG (2007). [The cellular location and significance of p38alpha/beta isoforms in the lumbar spinal cord of the bone cancer pain rats]. Zhonghua Yi Xue Za Zhi.

[76] Fitzsimmons BL, Zattoni M, Svensson CI, Steinauer J, Hua XY, Yaksh TL (2010). Role of spinal p38alpha and beta MAPK in inflammatory hyperalgesia and spinal COX-2 expression. Neuroreport.

