# Supplementary material for: Interleukin 33-mediated inhibition of A-type K+ channels induces sensory neuronal hyperexcitability and nociceptive behaviors in mice
Source: Theranostics. 2022 Feb 14;12(5):2232–47. doi: 10.7150/thno.69320 (PMC8899580; doi:10.7150/thno.69320)
Supplement: Supplementary file 1 — Supplementary figures. [file thnov12p2232s1.pdf]

Interleukin 33-mediated inhibition of A-type K^+ channels induces sensory neuronal hyperexcitability and nociceptive behaviours in mice

Yiru Wang, Xinyi Wang, Reifei Qi, Ying Lu, Yu Tao, Dongsheng Jiang, Yufang Sun, Xinghong Jiang, Chunfeng Liu, Yuan Zhang, Jin Tao

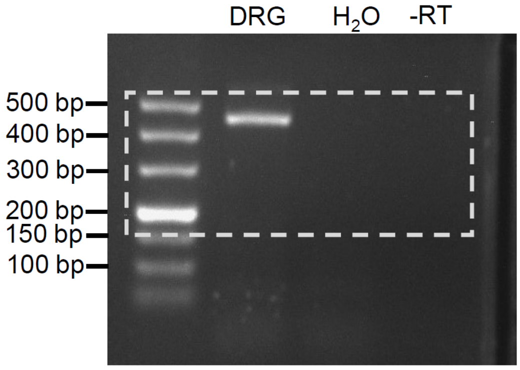

Figure S1. RT-PCR analysis of IL-33 transcripts in mouse DRGs. Shown are the full-length images of the blots presented in Figure 2A.

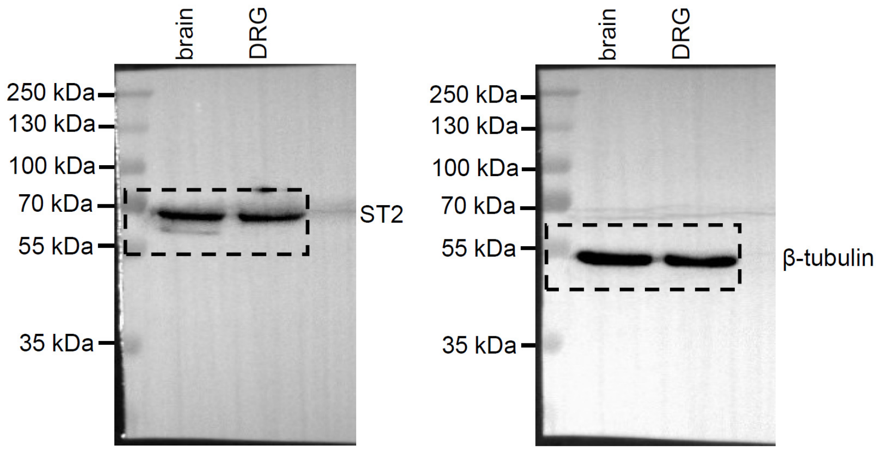

Figure S2. Abundance of ST2 in mouse TGs. Shown are the full-length images of the blots presented in Figure 2B. Blots are representative of three independent experiments with β-tubulin serving as loading control.

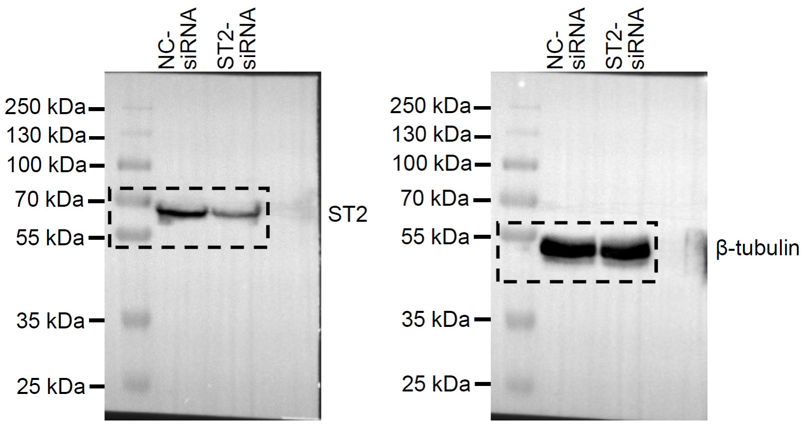

Figure S3. Knockdown of ST2 in mouse TGs. Shown are the full-length pictures of the blots presented in Figure 2E. The protein abundance of ST2 was measured using immunoblot analysis in NC-siRNA and ST2-siRNA groups. Blots are representative of three independent experiments with β-tubulin serving as loading control.

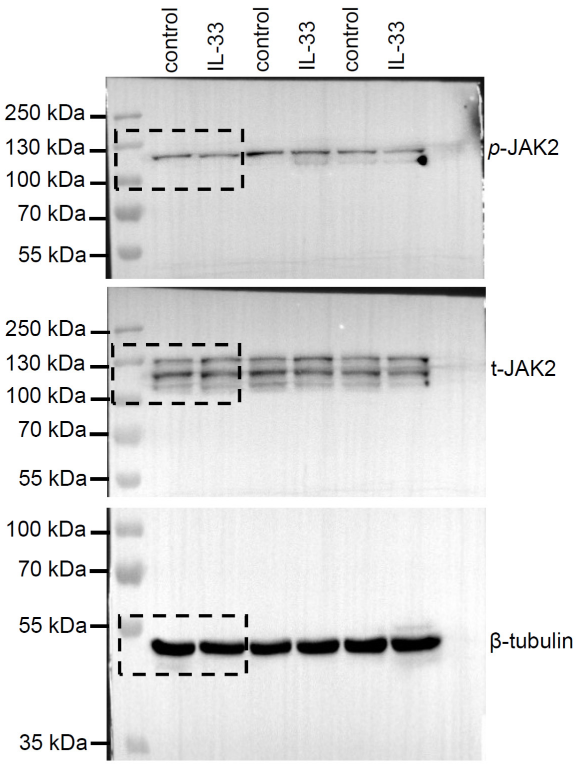

Figure S4. Change in the abundance of *p*-JAK2 induced by IL-33. Shown are the full-length pictures of the blots presented in Figure 3A. Blots are representative of three independent experiments with β-tubulin serving as loading control.

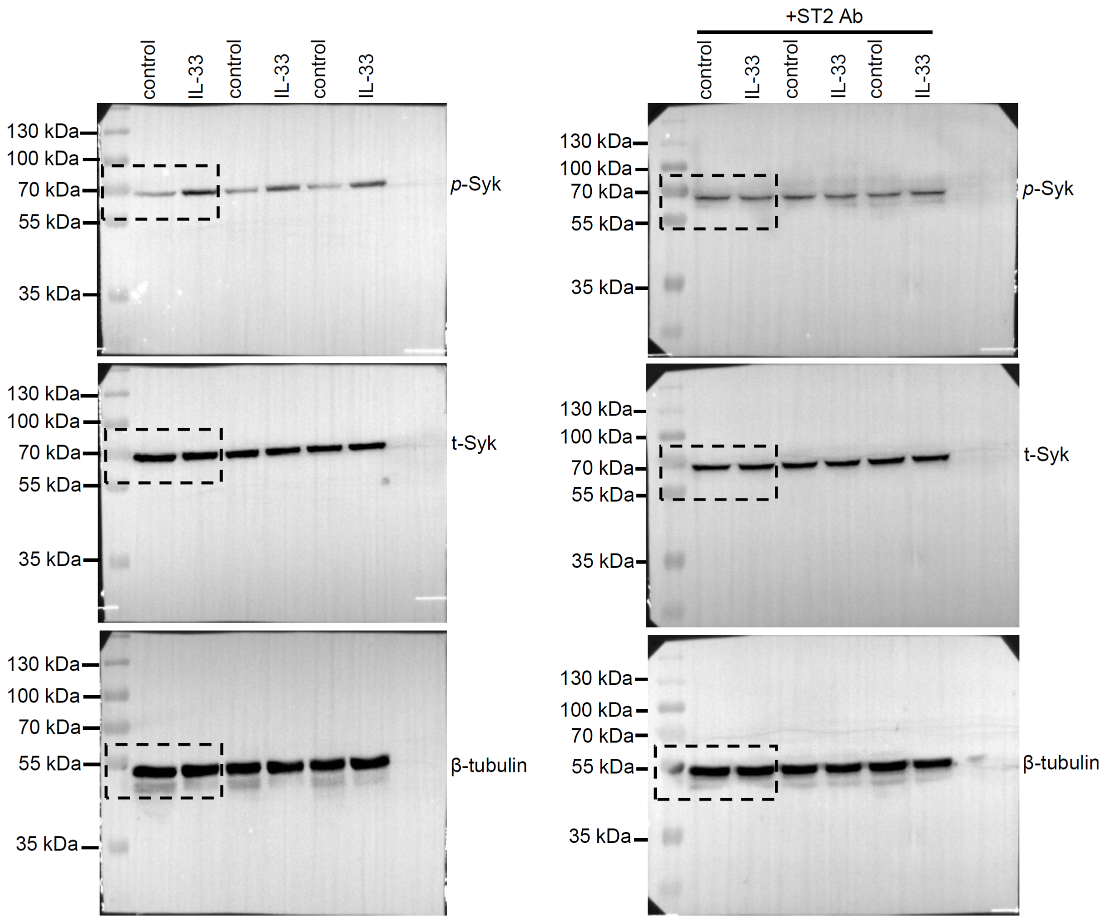

Figure S5. Change in the abundance of *p*-Syk induced by IL-33. Shown are the full-length pictures of the blots presented in Figure 3D. Pretreating cells with ST2 neutralizing antibody (ST2 Ab, 2 μg/mL) prevented the IL-33-induced increase in phospho-Syk expression. Blots are representative of three independent experiments with β-tubulin serving as loading control.

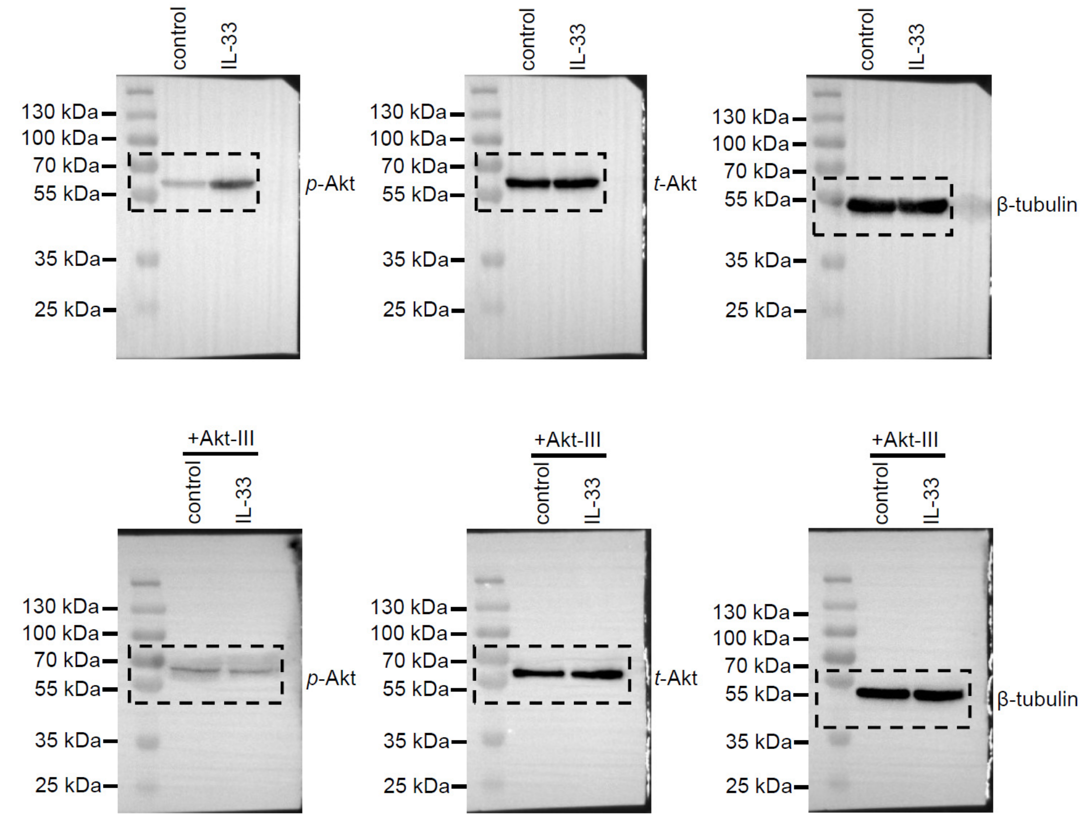

Figure S6. Change in the abundance of *p*-Akt induced by IL-33. Shown are the full-length pictures of the blots presented in Figure 4A. Pretreating cells with Akt inhibitor III (Akt-III, 10 μM) prevented the IL-33-induced increase in *p*-Akt expression. Blots are representative of three independent experiments with β-tubulin serving as loading control.

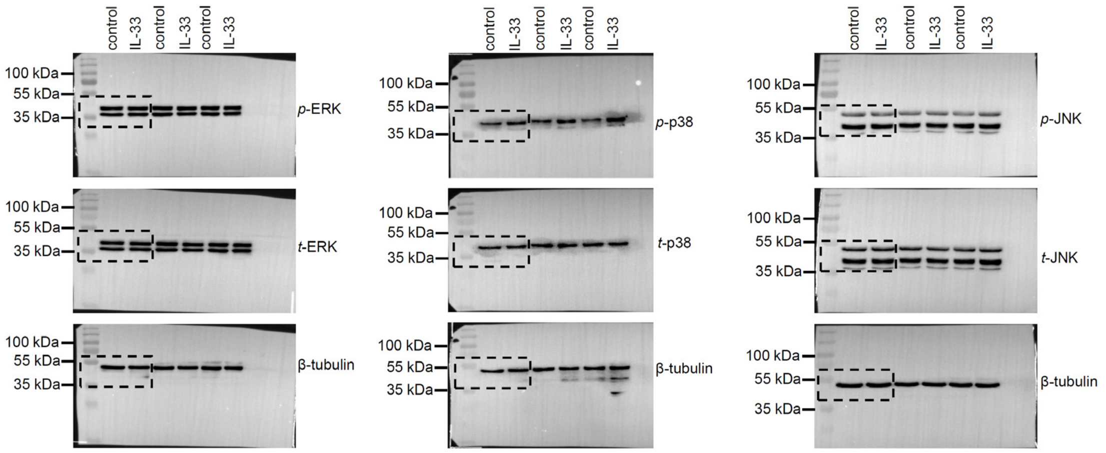

Figure S7. Change in the abundance of *p*-p38, *p*-JNK and *p*-ERK induced by IL-33. Shown are the full-length pictures of the blots presented in Figure 4F. Blots are representative of three independent experiments with β-tubulin serving as loading control.

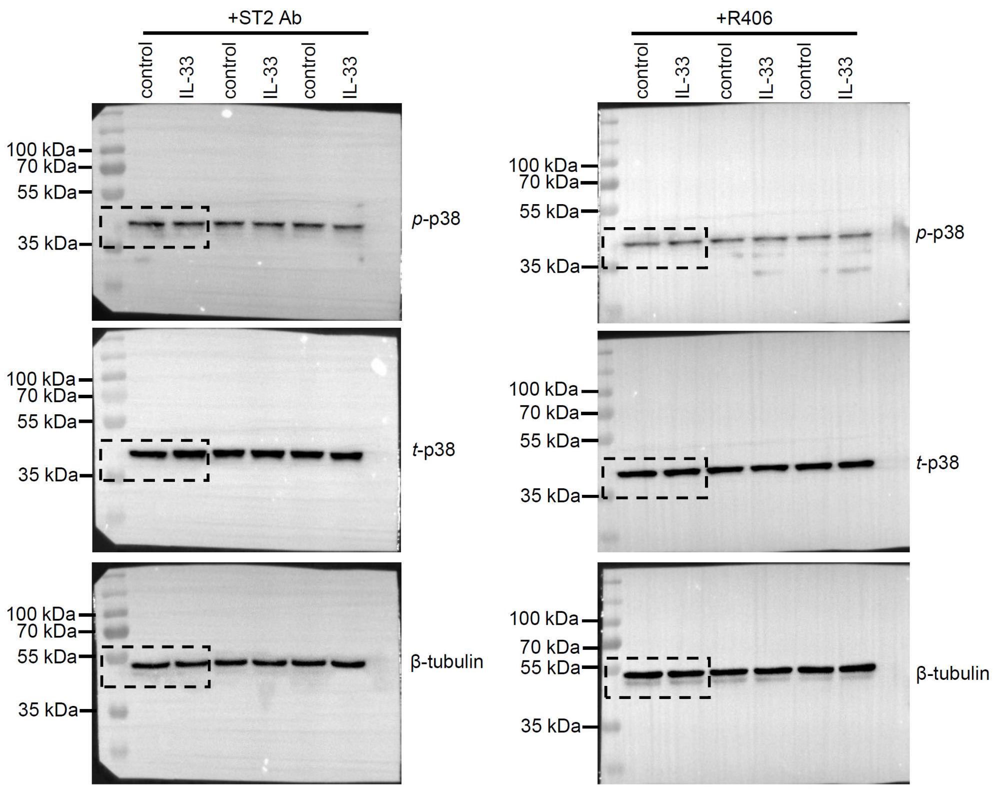

Figure S8. Change in the abundance of *p*-p38 induced by IL-33. Shown are the full-length pictures of the blots presented in Figure 4G. Pretreating cells with ST2 neutralizing antibody (ST2 Ab, 2 μg/mL) or R406 (1 μM) prevented the IL-33-induced increase in *p*-p38 expression. Blots are representative of three independent experiments with β-tubulin serving as loading control.

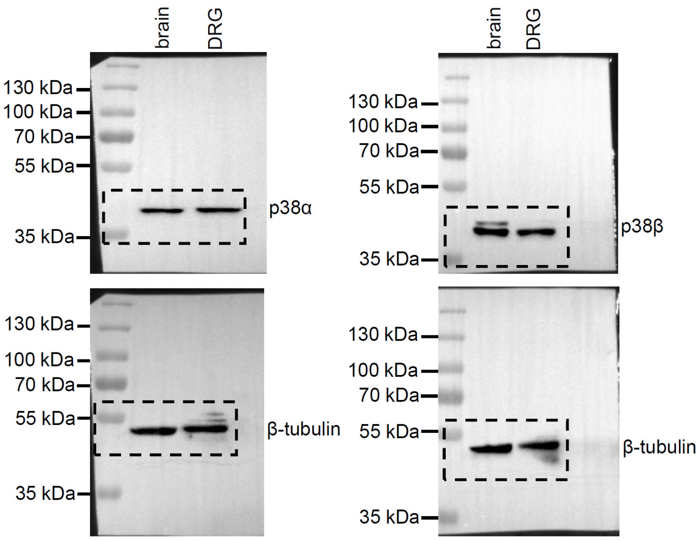

Figure S9. Abundance of p38α and p38β in mouse TGs. Shown are the full-length images of the blots presented in Figure 5A. Blots are representative of three independent experiments with β-tubulin serving as loading control.

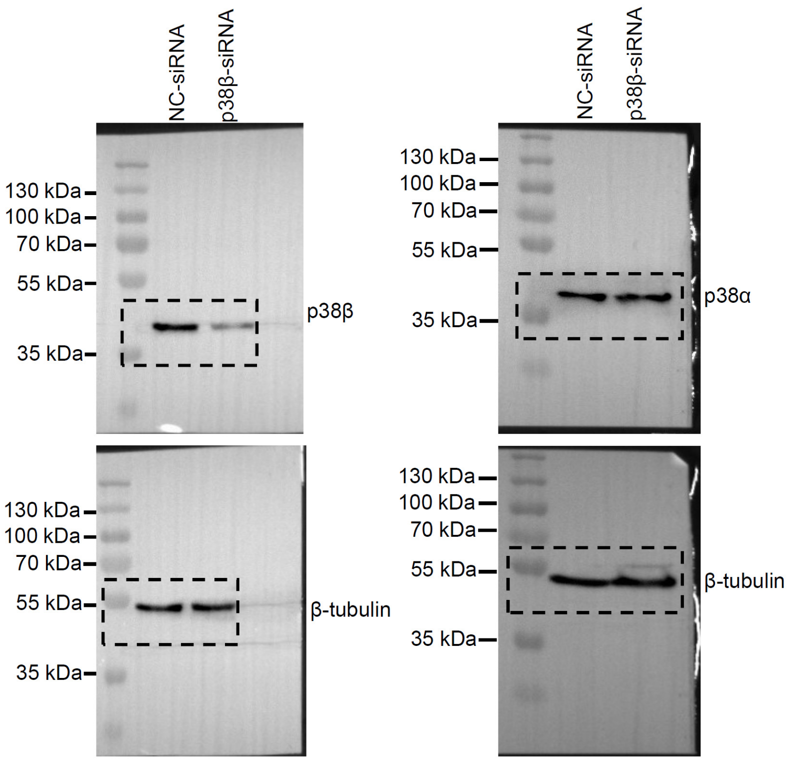

Figure S10. Knockdown of p38β in mouse TGs. Shown are the full-length pictures of the blots presented in Figure 5C. The protein abundance of p38α and p38β was measured using immunoblot analysis in NC-siRNA and p38β-siRNA groups. Blots are representative of three independent experiments with β-tubulin serving as loading control.

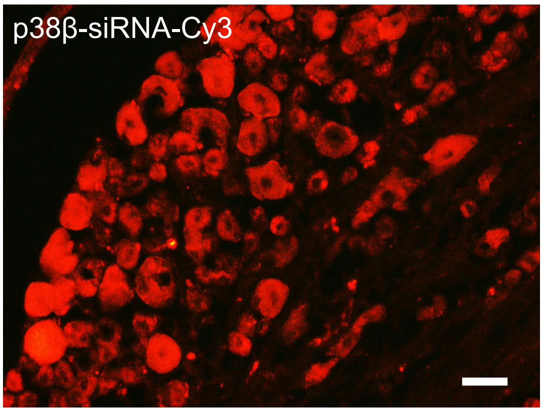

Figure S11. Representative image of Cy3 expression with red fluorescence in an intact DRG 3 days after lumbar intrathecal injection of a 5'-cholesteryl-modified and 2'-O-methyl-modified p38β siRNA labelled with Cy3. Scale bar, 50 μm.
